# Comparative Effects of Sleeve Gastrectomy vs. Roux-en-Y Gastric Bypass on Phase Angle and Bioelectrical Impedance Analysis Measures: A Systematic Review and Meta-Analysis

**DOI:** 10.3390/jcm13226784

**Published:** 2024-11-11

**Authors:** Julia Navarro-Marroco, Pilar Hernández-Sánchez, Desirée Victoria-Montesinos, Pablo Barcina-Pérez, Carmen Lucas-Abellán, Ana María García-Muñoz

**Affiliations:** 1 Faculty of Pharmacy and Nutrition, UCAM Universidad Católica San Antonio de Murcia, 30107 Murcia, Spain; jnavarro12@alu.ucam.edu (J.N.-M.); phsanchez@ucam.edu (P.H.-S.); clucas@ucam.edu (C.L.-A.); amgarcia13@ucam.edu (A.M.G.-M.); 2 Health Sciences Ph.D. Program, Universidad Católica de Murcia UCAM, Campus de los Jerónimos n°135, Guadalupe, 30107 Murcia, Spain

**Keywords:** obesity, bariatric surgery, phase angle, body composition, weight loss

## Abstract

**Background/Objectives:** The objective of this meta-analysis was to determine the impact of bariatric surgery on phase angle (PhA) and other bioimpedance measures among adults with obesity, comparing the effects of Roux-en-Y gastric bypass (RYGB) and sleeve gastrectomy (SG). **Methods:** A systematic review and meta-analysis were conducted following PRISMA guidelines, including studies up to May 2024 from MEDLINE, Scopus, Cochrane Library, and Web of Science. Eligible studies assessed PhA changes pre- and post-bariatric surgery in adults with BMI ≥ 30 kg/m^2^. Data on PhA, fat mass (FM), fat-free mass (FFM), body cell mass (BCM), weight, and BMI were extracted and analyzed. **Results:** Thirteen studies with a total of 1124 patients were included. Significant PhA reductions were observed at 6 months post-surgery (effect size: −1.00; 95% CI: −1.11 to −0.89; *p* < 0.001), with a more substantial reduction in RYGB patients compared to SG. FM and FFM decreased significantly at 12 months (FM: −27.58; 95% CI: −32.58 to −22.57; *p* < 0.001; FFM: −10.51; 95% CI: −12.81 to −8.94; *p* < 0.001). Weight and BMI showed marked reductions at 6 months (Weight: −31.42 kg; 95% CI: −37.28 to −25.26; *p* < 0.001; BMI: −11.39; 95% CI: −12.60 to −10.18; *p* < 0.001), with sustained decreases at 12 and 24 months. **Conclusions:** Bariatric surgery significantly reduces PhA, FM, FFM, weight, and BMI, with initial greater impacts observed in RYGB compared to SG. PhA shows potential as a marker for monitoring post-surgical recovery and nutritional status. Further long-term studies and standardized measurement protocols are recommended to optimize patient management.

## 1. Introduction

Obesity is a condition marked by excessive accumulation of body fat as well as metabolic, structural, and functional alterations. This leads to a state of chronic inflammation, which results in the emergence of complications such as type II diabetes, cardiovascular diseases, nutritional deficits, steatosis, psychological issues, and impaired immune function, among others [1]. Globally, up to 60% of the population faces weight-related issues and their complications [2]. In Spain, the prevalence is at 18.7% of the population, indicating that one in every five Spaniards suffers from this condition, leading to a 9.7% increase in public healthcare spending [3]. It has been found that as age increases and socioeconomic resources decrease, the risk of obesity rises along with the risk of sarcopenia in adults, reducing quality of life and metabolic health, as well as increasing mortality, admissions, hospitalizations, and the onset of pathophysiological comorbidities [3,4,5].

In patients with a BMI > 40 kg/m^2^ or with a BMI between 30 and 40 kg/m^2^, but with one or more associated comorbidities such as cardiovascular risk, type II diabetes mellitus, apnea, hypertension, gastroesophageal reflux disease, and bariatric surgery is indicated. This procedure aims at weight loss through modifications of the digestive system, reducing the intake or absorption of nutrients. This is achieved through various techniques such as adjustable gastric banding, sleeve gastrectomy, or gastric bypass [6]. Despite the risks, as with all surgeries, the use of bariatric surgery in obese patients has reported benefits such as sustained weight loss, at least 50% of the excess, and more successful maintenance over time, as well as improvement or remission of comorbidities like insulin resistance, type 2 diabetes mellitus, steatosis, etc., making it currently the most effective treatment [6,7,8].

The phase angle (PhA), a biomarker obtained through bioimpedance by measuring the resistance and reactance that tissues present to the flow of current, represents the state of hydration and body cell mass. PhA is closely related to the percentage of fat mass (FM), total body water (TBW), and the ratio of extracellular water (ECW) to TBW, parameters that are altered in obesity and are predictors of malnutrition and clinical outcomes [9,10]. It is noted that some markers traditionally used for evaluating bariatric patients have limitations mainly related to the amount of fat-free mass (FFM), muscle mass, and nutritional status. PhA is a value that considers the ratio of intracellular to extracellular water (ICW/ECW) as well as the amount of ECW present in adipose tissue, making its assessment potentially useful in improving the analysis of patient body composition. PhA shows positive correlations with muscle markers, albumin, and total proteins and negative correlations with the percentage of fat mass, interesting data when assessing the physical and functional state of the patient. In obese patients, candidates for bariatric surgery, PhA can be useful as a marker of catabolism and systemic inflammation, since it has been shown to relate to levels of markers such as IL-6 and CRP, among others [10]. It can also serve as a follow-up value during weight loss or as a preoperative value, possibly screening more efficiently those patients at higher or lower risk of comorbidities, candidates for surgery, or candidates for a multidisciplinary approach prior to intervention. A study conducted by Cancello et al. determines that PhA can be an important predictive marker of the early stages of malnutrition in these patients, as well as a valuable tool in monitoring successful weight loss by controlling parameters such as FM and FFM, the presence and development or improvement of associated comorbidities, and cellular status [10]. Moreover, a recent systematic review by Di Vincenzo et al. concludes that PhA is an optimal value for assessing the nutritional status and muscle quality of patients; its monitoring during and after bariatric surgery can help to have a more complete view of changes in body composition parameters like body cell mass (BCM), fat mass, fat-free mass, and fluid distribution [11].

Therefore, considering the high variability in PhA response to different bariatric interventions and the need for more extensive research to determine protocols, strategies, and cutoff points for PhA in such patients, the main goal of this meta-analysis was to explore the pre- and post-surgery PhA values and their increase or decrease depending on the changes experienced in parameters such as FM, FFM, and BCM during weight loss following bariatric intervention. This analysis aims to elucidate the potential of PhA as a predictive and monitoring biomarker in the context of bariatric surgery, highlighting its significance for preoperative evaluation and postoperative monitoring of patients.

## 2. Materials and Methods

### 2.1. Search Strategy and Selection of Studies

The present review and meta-analysis were conducted in accordance with the PRISMA (preferred reporting items for systematic reviews and meta-analyses) guidelines [12] and following the Cochrane Collaboration’s Manual for Systematic Reviews of Interventions [13]. The protocol was registered in PROSPERO with the registry number CRD42023489544.

### 2.2. Eligibility Criteria

Eligible studies were those that evaluated the impact of bariatric surgery on the phase angle in adult participants with obesity. The search and selection of studies were performed by two independent reviewers (J.N.-M. and D.V.-M.) who examined titles and abstracts according to the following PICOS criteria:

Participants: Inclusion criteria focused on adults aged 18 years or older diagnosed with obesity, defined as a body mass index (BMI) ≥30 kg/m^2^. Studies involving participants with specific subtypes of obesity, such as class II (BMI 35.0–39.9 kg/m^2^) and class III (BMI ≥ 40 kg/m^2^) obesity, were particularly sought to understand the effects of bariatric surgery across a spectrum of obesity severity.

Intervention: The interventions of interest were bariatric surgery procedures, including but not limited to Roux-en-Y gastric bypass (RYGB) and sleeve gastrectomy (SG). Studies that provided detailed descriptions of the surgical technique, perioperative care, and follow-up protocols were included to ensure comprehensive analysis of the surgical impact on phase angle measurements.

Comparison: The groups compared were patients who underwent SG and those who received RYGB, allowing for a direct evaluation of surgical interventions on phase angle and other BIA measures. This comparison focuses specifically on the differential impacts of these two primary bariatric surgery techniques rather than comparing surgical outcomes to non-surgical obesity management strategies.

Outcome: The primary outcome was changes in phase angle measurements post-bariatric surgery, as assessed by BIA. Studies were included if they reported PhA values both pre- and post-surgery, enabling the evaluation of surgery-related changes over time. Secondary outcomes of interest included changes in body composition (e.g., fat mass and lean body mass) and nutritional status indicators, as these could influence PhA measurements.

Study Design: We considered randomized controlled trials (RCTs), non-randomized clinical trials, cohort studies, case-control studies, and cross-sectional studies, ensuring a broad capture of available evidence across different research designs.

Exclusion Criteria: Studies were excluded if they were review articles, editorials, or case reports. Additionally, studies not reporting specific outcomes related to PhA or lacking pre-surgical baseline measurements were omitted. Studies published in languages other than English were also excluded due to potential challenges in accurate translation and interpretation.

### 2.3. Information Sources and Search Strategy

A systematic search was conducted in MEDLINE (via PubMed), Scopus, Cochrane Library, and Web of Science databases from inception until 2 May 2024. The search strategy combined terms related to obesity (“obesity”, “bariatric surgery”, “gastric bypass”, “sleeve gastrectomy”) with terms specific to the outcome of interest (“phase angle”, “bioelectrical impedance analysis”). The complete search strategy is presented in Table 1.

### 2.4. Data Collection Process and Data Items

For data extraction, an initial phase was conducted by one author (J.N.-M.), and subsequently, the information extracted was cross-checked point by point against the original articles by a second author (D.V.-M.). In the event of any discrepancies between these two researchers, a third researcher (A.M.G.-M.) reviewed the information.

Data were extracted on study reference, country of intervention, and participant characteristics (total sample size, intervention and control groups, sex ratio, age range, mean age, and baseline weight status), intervention duration, type of bariatric procedure, setting, and outcome information related to PhA measurements.

### 2.5. Study Risk of Bias Assessment

To assess the risk of bias in non-randomized studies included in this meta-analysis, the ROBINS-I (risk of bias in non-randomized studies of interventions) tool will be employed [14]. ROBINS-I is designed to evaluate bias due to confounding, selection of participants, classification of interventions, deviations from intended interventions, missing data, measurement of outcomes, and selection of the reported result. Each domain is critically analyzed, and the study’s risk of bias is categorized as “low”, “moderate”, “serious”, or “critical” for each domain, facilitating a comprehensive bias assessment.

The bias risk was evaluated independently by two reviewers (D.V.-M. and A.M.G.-M.). In cases of disagreement between them, a third reviewer (J.N.-M.) was consulted to achieve consensus.

### 2.6. Synthesis Methods

The main effect size of bariatric surgery on PhA was quantified using standardized mean difference (SMD). Meta-analyses were performed using a random-effects model (DerSimonian and Laird method) to calculate the pooled estimate of effect size and 95% confidence intervals (CIs) for the impact of bariatric surgery on PhA and secondary variables. Additionally, a subgroup analysis was performed based on the surgical technique employed (RYGB or SG). This analysis allowed exploration of whether the overall effect varied according to the type of surgical intervention. Standard deviations were derived from standard errors or confidence intervals as required. In cases where necessary, data were extracted from graphs using WebPlotDigitizer (version 4.5, Ankit Rohatgi, 2021), ensuring accuracy through double-checking by independent reviewers.

Transformation of results was conducted in accordance with the guidelines provided in the Cochrane Handbook [13].

Forest plots were created to visually represent the data, complete with 95% confidence intervals (CI). Each study’s effect size was calculated and categorized as small (0–0.20), medium (>0.20 to 0.50), or large (>0.50). Negative estimated effect values were interpreted as reductions for all variables analyzed.

Heterogeneity was assessed using the I^2^ statistic [15]. The I^2^ values were classified into four categories: not significant (<40%), moderate (40–60%), substantial (60–75%), and considerable (75–100%) [16].

In order to evaluate the consistency of the overall results, a leave-one-out sensitivity analysis was performed. This method consists of excluding each individual study from the meta-analysis and recalculating the overall effect size at each iteration. This approach allowed identification of whether any study had a disproportionate impact on the overall result.

To assess the presence of reporting bias, including publication bias and small study effects, the Luis Furuya–Kanamori (LFK) index together with the Doi plot was used for a visual and quantitative assessment of asymmetry in the meta-analysis results [17]. The LFK index quantifies the skewness of the Doi plot, offering a numeric value to assess potential publication bias. An LFK index of 0 indicates no asymmetry, suggesting an absence of publication bias. Values between 1 and 2 indicate minor asymmetry, which may suggest moderate publication bias, while values of 2 or more indicate major asymmetry, signalling significant concerns for publication bias. This approach enhances the rigor of the reporting bias assessment process, helping to identify asymmetries that could distort the estimated effects reported in the meta-analysis and ensuring that conclusions are more robust and credible.

All statistical analyses were performed using Stata software (version 16.1; StataCorp, College Station, TX, USA), with a significance threshold set at *p* < 0.05.

## 3. Results

### 3.1. Study Selection

These meta-analyses synthesize the findings from a comprehensive review of studies investigating the effects of bariatric surgery on PhA values among adults diagnosed with obesity. Bariatric surgery, encompassing various procedures such as RYGB and SG, has been increasingly recognized not only for its capacity to achieve significant weight loss but also for its potential to modify body composition and cellular integrity, as indicated by PhA measurements. PhA, derived from bioelectrical impedance analysis, serves as a non-invasive marker of cellular health and nutritional status, reflecting the integrity of cell membranes and the distribution of body fluids. Given the prevalence of obesity worldwide and the growing utilization of bariatric surgery as a treatment modality, understanding its comprehensive impacts, including alterations in PhA, is of paramount importance.

The included studies span several countries and collectively encompass a diverse patient population, offering a broad perspective on the outcomes of bariatric surgery beyond traditional measures of success such as weight loss. By evaluating changes in PhA, this analysis aims to shed light on the potential physiological and metabolic benefits of bariatric surgery that extend beyond mere reductions in BMI. Through rigorous selection criteria, our analysis focused on studies that provided clear and measurable outcomes regarding PhA variations pre- and post-bariatric surgery, allowing us to critically assess the evidence on this emerging marker of health improvement in the obese population.

**Figure 1:**
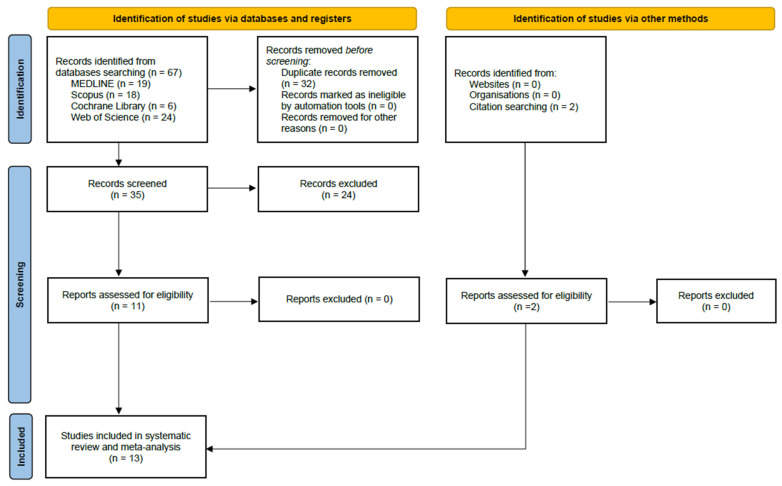
Flow diagram for preferred reporting items for systematic reviews and meta-analyses (PRISMA).

A total of 67 records were identified through database searches (Figure 1). After duplicates were removed, 35 records were left for further evaluation. Screening based on titles and abstracts resulted in the exclusion of 24 publications as they did not align with the specific objectives of this review. Finally, 13 studies were included in the systematic review and meta-analysis.

### 3.2. Study Characteristics

The main characteristics of the 13 studies included in this systematic review and meta-analysis [18,19,20,21,22,23,24,25,26,27,28,29] are detailed in Table 2.

In meta-analyses focusing on the impact of bariatric surgery on obesity and PhA, the compilation of 13 studies presents a rich tapestry of research conducted across diverse global locales, including Germany (5 studies; [18,20,21,22,30]), Brazil (6 studies; [19,23,25,26,28,29]), Iran [24], and Spain [27]. These studies collectively examined 1124 patients. The distribution between the two primary types of bariatric surgeries investigated was as follows: 189 patients underwent SG, and 935 patients underwent RYGB. The average participant profile across these studies illustrates a middle-aged demographic, where 83.6% were women with a mean age around 42 years (41.89 ± 8.8), grappling with severe obesity as evidenced by an initial average BMI of 44.6 ± 6.3 kg/m^2^.

The studies explored various dimensions of the surgical impact, from body composition changes to nutritional status, utilizing a range of variables including PhA, BMI, weight, FM, FFM, lean body mass (LBM), and BCM. The duration of these studies varied, with follow-ups ranging from 6 to 24 months post-surgery, providing a longitudinal view of the patients’ progress.

### 3.3. Risk of Bias in Included Studies

The risk of bias assessment for the non-randomized studies was conducted using the ROBINS-I tool. The predominant risk of bias among the studies was moderate, primarily due to the inherent challenges in addressing confounding variables. Studies such as those by Vassilev et al. [18], Florencio et al. [19], Otto et al. [20], Gerken et al. [21], Venancio et al. [23], Vassilev et al. [22], Golzarand et al. [24], Koehler et al. [25], Bortoli et al. [26], and Teixeira et al. [28] exhibited moderate risk primarily due to potential residual confounding. These studies often faced difficulties in fully controlling for all confounding factors, which could impact the observed outcomes. Confounding variables, such as baseline health status, socio-economic factors, and pre-existing conditions, were not always adequately accounted for, potentially leading to biased effect estimates.

In addition to confounding, the selection of participants was another source of moderate bias in these studies. Specific inclusion and exclusion criteria might have introduced selection bias by limiting the generalizability of the findings to broader populations. For instance, studies might have excluded participants with certain comorbidities or those outside a specific age range, which could skew the results.

On the other hand, Martínez et al. [27], Renata Manoel et al. [29], and Friedrich [30] demonstrated a lower overall risk of bias. These studies managed to handle confounding factors more effectively through comprehensive statistical adjustments and robust study designs that minimized the impact of these variables. Additionally, they ensured a more representative participant selection, enhancing the external validity of their findings.

The domains with the lowest risk of bias across all studies were “Bias in the classification of interventions” and “Bias due to deviations from intended interventions”. These domains were well handled because the studies provided clear definitions and consistent applications of the interventions. Detailed protocols and adherence to planned interventions reduced the likelihood of bias from deviations. Furthermore, the studies generally dealt with missing data appropriately by employing techniques such as multiple imputation or sensitivity analyses to ensure that the missing data did not significantly affect the results.

Measurement of outcomes also exhibited a low risk of bias, as most studies employed validated measurement tools and ensured that outcome assessors were blinded to intervention status. This blinding reduced the potential for measurement bias and increased the reliability of the outcome data.

**Figure 2:**
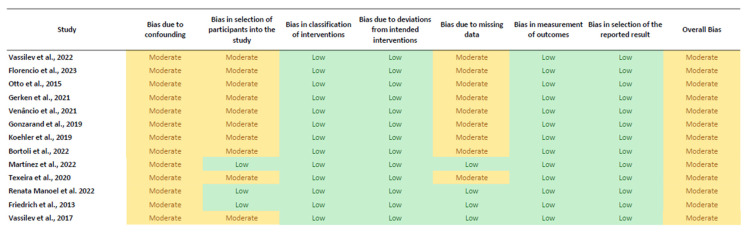
Risk of Bias Assessment using ROBINS-I for the Included Intervention Studies. Green indicates low risk of bias and yellow indicates moderate risk of bias [18,19,20,21,22,23,24,25,26,27,28,29,30].

These results can be seen in Figure 2.

### 3.4. Results of Syntheses

#### 3.4.1. Phase Angle

Across the board, the studies reviewed present a compelling narrative of PhA alteration following bariatric surgery, highlighting the intricate relationship between weight loss, body composition changes, and cellular health. For instance, Vassilev et al. [18] observed a notable reduction in PhA from 6.38 to 5.7 at 6 months post-surgery, paralleling significant weight loss and BMI reduction. This trend is mirrored in the findings of Florencio et al. [19] and Otto et al. [20], where phase angle reductions to 3.7 and 5.4, respectively, underscore the physiological shifts occurring within the body’s cellular environment in response to substantial fat mass loss and changes in body composition.

Moreover, Gerken et al. [21] provide a differentiated perspective by comparing outcomes between SG and RYGB patients, with PhA changes offering a lens through which the metabolic and cellular impact of different surgical approaches can be assessed. While the phase angle reduction reflects a universal response to the acute phase of postoperative weight loss, the degree of change varies, suggesting individual variability in cellular adaptation to the metabolic changes induced by surgery.

The analysis extends to studies like those conducted by Venâncio et al. [23], Golzarand et al. [24], Friedrich et al. [30], Manoel et al. [29], and Martínez et al. [27], each contributing to a broader understanding of the temporal dynamics of PhA adjustments over time. Venâncio et al. [23] demonstrated that RYGB might be more effective in attenuating oxidative damage after 6 months, with changes in body impedance analysis parameters inversely correlated with protein oxidative damage. Golzarand et al. [24] noted that the decrease in PhA postoperatively reflects a reduction in lean body mass or an increase in fat mass, while Martínez et al. [27] highlighted the continuous and substantial loss of FFM after bariatric surgery. These studies collectively illustrate a gradual stabilization or modest improvement in PhA values beyond the initial post-surgery months, hinting at a potential recovery or enhancement of cellular health as the body adjusts to its new physiological state post-weight loss.

The PhA, as an indicator of cellular integrity and nutritional status, shows increases or stabilization in some studies at the 6-month interval. However, long-term evidence on PhA changes is limited, highlighting a research gap. Notably, for the studies providing 24-month data, like Teixeira et al. [28], there’s an intriguing hint that improvements in PhA may reflect sustained or even enhanced cellular health and nutritional status over time. However, with only a few data points available at this mark, the conclusions remain tentative. Florencio et al. [19] and Martínez et al. [27] contribute to this long-term perspective, offering insights into the durability of phase angle improvements as indicative of ongoing adjustments in body composition and cellular health.

Regarding PhA, eleven studies presented PhA values at six months post-bariatric intervention [18,19,20,21,23,24,25,26,27,29,30], six studies continued the measurement at 12 months [21,22,26,27,29,30] and three studies extended their assessments to 24 months [21,27,28].

**Figure 3:**
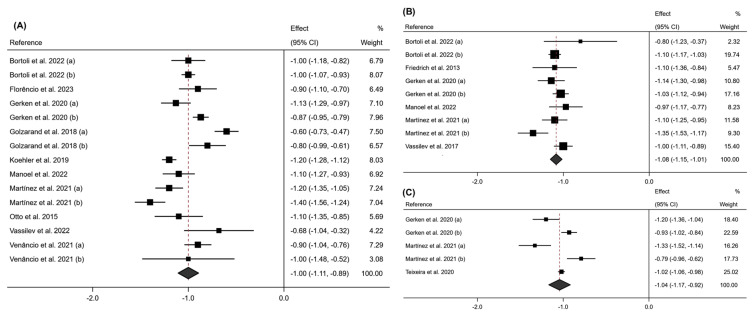
Forest plot detailing the effect size and 95% confidence intervals (CI) for the effect of surgical intervention on PhA at (**A**) 6 months post-intervention; (**B**) 12 months post-intervention; (**C**) 24 months post-intervention. (a) Sleeve Gastrectomy; (b) Roux-en-Y Gastric Bypass [18,19,20,21,22,23,24,25,26,27,28,29].

After evaluating the studies for the variable PhA at 6 months post-intervention, a significant decrease was observed for this variable (effect size: −1.00; 95% CI: −1.11 to −0.89; *p* < 0.001) (I^2^ = 88.4%; 95% CI: 58.9% to 94.7%; *p* < 0.001). This variable continued to decline at 12 post-intervention (effect size: −1.09; 95% CI: −1.15 to −1.02; *p* < 0.001) (I^2^ = 49.8%; 95% CI: 0.0% to 78.2%; *p* < 0.04) and at 24 months post-intervention (effect size: −1.04; 95% CI: −1.17 to −0.92; *p* < 0.001) (I^2^ = 85.0%; 95% CI: 0.0% to 95.5%; *p* < 0.001). These results are shown in Figure 3.

The LFK index in the Doi plots showed lower asymmetry (LFK index = −1.75) for PhA at 6 months of intervention follow-up, at 12 months (LFK index = −1.97) and at 24 months (LFK index = −1.33). These are shown in Figure S1 of the Supplementary Materials.

The leave-one-out sensitivity analysis for the PhA showed no significant change in the overall effect sizes (variations remained within an acceptable range (±10%)). These results can be found in Figure S2 of Supplementary Materials.

**Figure 4:**
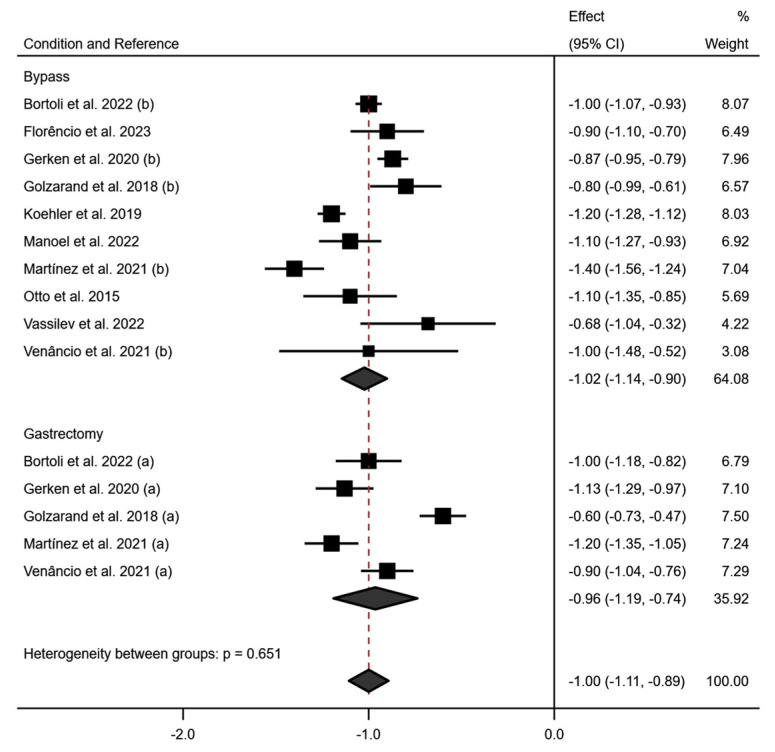
Forest plot detailing the effect size and 95% confidence intervals (CI) for the effect of each type of surgical intervention on PhA at 6 months post-intervention. (a) Sleeve Gastrectomy; (b) Roux-en-Y Gastric Bypass [18,19,20,21,22,23,24,25,26,27,29].

Figure 4 shows the subgroup analysis according to the surgical technique used for PhA at 6 months. Specifically, this analysis showed a variation in the decrease in PhA according to the type of technique used, with a greater decrease in PhA in the RYGB technique (−1.02; 95% CI −1.14 to −0.90; *p* < 0.001) (I^2^ = 86.5%; *p* < 0.001) than in Gastrectomy (−0.96; 95% CI −1.19 to −0.74) (I^2^ = 91.5%; *p* < 0.001) at 6 months postoperatively.

At 12 months post-surgery, it was observed that the decrease in PhA values in RYGB and SG are similar (RYGB: −1.08; 95% CI −1.18 to −0.99 (I^2^ = 70.5%; *p* < 0.009); SG: −1.10; 95% CI −1.20 to −1.00 (I^2^ = 0.0%; *p* = 0.541); while at 24 months post-surgery, PhA in patients operated with RYGB technique increases and in the SG technique continues to decrease (RYGB: −0.94; 95% CI −1.05 to −0.82 (I^2^ = 78.5%; *p* < 0.009); SG: −1.25; 95% CI −1.38 to −1.13 (I^2^ = 8.0%; *p* = 0.297). These results are shown in Supplementary Figure S3.

#### 3.4.2. Fat Mass and Body Composition

This analysis highlights the transformative effects of bariatric procedures not just in terms of weight loss but, more importantly, on improving overall body composition, a critical factor in reducing obesity-related health risks.

For instance, the study by Gerken et al. [21] in Germany on SG patients showed a remarkable reduction in FM from an initial average of 48.33 kg to 41.69 kg at 6 months, alongside improvements in BCM, LBM, and FFM, underscoring the surgery’s efficacy in enhancing metabolic health. Similarly, the cohort in Golzarand et al. [24] from Iran exhibited significant changes in body composition post-surgery, with FM decreasing alongside notable increases in LBM and FFM, indicative of a shift towards a healthier physiological state.

These findings are echoed across other studies in our analysis, spanning countries like Brazil and Spain, with both SG and RYGB procedures demonstrating substantial reductions in fat mass percentages and increases in lean mass measures. Notably, the study by Vassilev et al. [18] detailed a decrease in fat mass alongside a reduction in BMI, showcasing the dual benefits of bariatric surgery in managing both visible and visceral obesity markers. Additionally, the study by Martínez et al. [27] in Spain emphasized the continuous and substantial loss of FFM after bariatric surgery, amounting to about 21.71% of total weight loss 24 months post-surgery, highlighting the nuanced effects of surgery on body composition.

However, variations exist in the magnitude of these changes, likely attributable to differences in surgical techniques, post-operative care, and patient adherence to dietary recommendations. While most studies report outcomes at 6 months, extending the follow-up period, as seen in Teixeira et al. [28] with a 24-month horizon, reveals the sustained impact of surgery on body composition, suggesting long-term benefits that extend well beyond initial weight loss. Furthermore, the studies underscore the improvement in other health parameters beyond mere weight metrics, such as reductions in FM and alterations in body composition, indicating a holistic improvement in patient health following bariatric surgery.

Regarding body composition, studies like Gerken et al. [21] and Martínez et al. [27] provide valuable data on FM decrease and changes in FFM at the 6-month mark, with Martínez et al. [27] extending the follow-up period to 24 months. This long-term information highlights substantial improvements in body composition, likely contributing to the observed long-term health benefits.

**Figure 5:**
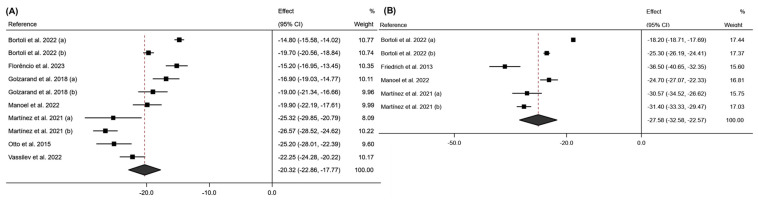
Forest plot detailing the effect size and 95% confidence intervals (CI) for the effect of surgical intervention on fat mass at (**A**) 6 months post-intervention; (**B**) 12 months post-intervention. (a) Sleeve Gastrectomy; (b) Roux-en-Y Gastric Bypass [18,19,20,22,23,24,26,27,29].

The meta-analysis conducted for the FM variable showed a significant decrease in this variable at 6 months post-intervention (effect size: −20.32; 95% CI: −22.86 to −17.77; *p* < 0.001) (I^2^ = 95.9%; 95% CI: 70.1% to 98.5%; *p* < 0.001), with this decrease becoming more pronounced at 12 months post-intervention (effect size: −27.58; 95% CI: −32.58 to −22.57; *p* < 0.001) (I^2^ = 98.8%; 95% CI: 38.0% to 99.7%; *p* < 0.001). These results can be seen in Figure 5.

**Figure 6:**
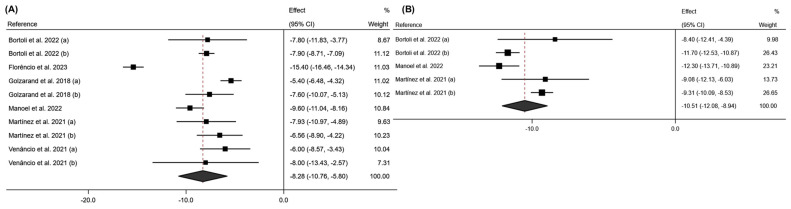
Forest plot detailing the effect size and 95% confidence intervals (CI) for the effect of surgical intervention on free fat mass at (**A**) 6 months post-intervention; (**B**) 12 months post-intervention. (a) Sleeve Gastrectomy; (b) Roux-en-Y Gastric Bypass [19,23,24,26,27,29].

Regarding FFM, a similar trend was observed in Figure 6, with a greater loss of LBM at 12 months post-intervention ((12 months: Effect size: −10.51; 95% CI: −12.81 to −8.94; *p* < 0.001) (I^2^ = 84.0%; 95% CI: 0.0% to 95.3%; *p* < 0.001); (6 months: Effect size: −8.28; 95% CI: −10.76 to −5.80; *p* < 0.001) (I^2^ = 95.5%; 95% CI: 69.8% to 98.3%; *p* < 0.001)).

According to the type of surgical technique employed, it was observed that patients who underwent the RYGB technique experienced a more pronounced decrease in FM and FFM at 6 months post-intervention compared to those who underwent SG (FM RYGB: Effect size: −21.06; 95% CI: −23.71 to −18.41; *p* < 0.001) (I^2^ = 93.4%; *p* < 0.001 vs. FM SG: Effect size: −18.32; 95% CI: −22.42 to −14.21; *p* < 0.001) (I^2^ = 91.1%; *p* < 0.001). However, this trend reverses at 12 months of follow-up, with a greater decrease in FM observed in patients who underwent SG (FM RYGB: Effect size: −27.18; 95% CI: −31.08 to −23.16; *p* < 0.001) (I^2^ = 94.0%; *p* < 0.001 vs. FM SG: Effect size: −28.30; 95% CI: −40.67 to −15.92; *p* < 0.001) (I^2^ = 98.2%; *p* < 0.001). These results can be seen in Figure S4 of the Supplementary Materials.

For the FFM variable, a more pronounced decrease was observed in patients who underwent RYGB compared to those who underwent SG at both 6 months and 12 months post-intervention. These results can be seen in Figure S5 of the Supplementary Materials.

The FM variable at 12 months of follow-up and FFM at 6 and 12 months of follow-up showed minor asymmetry (LFK index FFM 12 months = −1.54) as at 12 months (LFK index FM 6 months = −1.19; LFK index FFM 12 months = −1.50); whereas the FM variable at 6 months showed major asymmetry (LFK index FM 6 months = −2.07). The Doi plots and LFK indices can be seen in Figure S6 of the Supplementary Materials.

**Figure 7:**
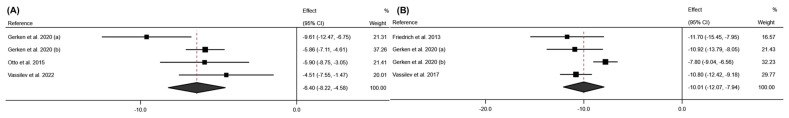
Forest plot detailing the effect size and 95% confidence intervals (CI) for the effect of surgical intervention on lean body mass at (**A**) 6 months post-intervention; (**B**) 12 months post-intervention. (a) Sleeve Gastrectomy; (b) Roux-en-Y Gastric Bypass [18,20,21,22,30].

These results are consistent with those observed in the LBM variable (Figure 7). After 6 months of intervention, an average effect of −6.40 was observed for this variable (95% CI: −8.22 to −4.58; *p* < 0.001; I^2^ = 56.9%; 95% CI: 0.0% to 86.6%; *p* = 0.073). At 12 months post-intervention, a further decrease in this variable was observed, with an average effect of −10.01 (95% CI: −12.07 to −7.94; *p* < 0.001; I^2^ = 74.2%; 95% CI: 0.0% to 92.5%; *p* < 0.009).

The SG surgical technique showed a greater average decrease in this variable compared to the RYGB technique at both 6- and 12-months post-intervention. At 6 months post-SG surgery, an average effect size of −9.61 was observed (95% CI: −12.47 to −6.75; *p* < 0.001), while at 12 months, the average effect size was −11.21 (95% CI: −13.48 to −8.93; *p* < 0.001). These results can be seen in Figure S7 of the Supplementary Materials.

Finally, major asymmetry was observed at both 6 months and 12 months post-intervention for this variable (LFK index LBM 6 months = −2.03; LFK index LBM 12 months = −2.83). These results can be seen in Figure S8 of the Supplementary Materials.

A leave-one-out sensitivity analysis was performed to assess the robustness of the results for the variables FM, FFM, and LBM. In the case of FM, the exclusion of each individual study did not lead to significant changes in the overall effect size, suggesting that the results are stable and not unduly influenced by any study. However, for the FFM and LBM variables, exclusion of certain studies resulted in a greater than 10% change in the overall effect size. In FFM, the exclusion of the Florêncio et al. study (effect size: −7.37; 95% CI: −8.52 to −6.21) had a considerable impact, while in LBM, the exclusion of the Gerken et al. study (a) [21] also showed a noticeable change in effect (effect size: −5.70; 95% CI: −6.77 to −4.68). These results can be found in Figure S9 in the Supplementary Materials.

#### 3.4.3. Body Cell Mass

The evaluation of BCM post-surgery provides a detailed view of changes in metabolically active tissue and helps identify potential nutritional deficits and muscle mass losses that can compromise recovery and patient quality of life. Specifically, monitoring BCM in post-RYGB and SG patients allows healthcare professionals to assess the effectiveness of the surgical intervention and adjust follow-up and nutrition strategies to ensure patients maintain a healthy cell mass. Preserving BCM is crucial for maintaining basal metabolism and physical functionality, which directly influences the long-term outcomes of bariatric surgery.

Five studies evaluated BCM at 6 months post-surgery [18,20,21,24,29], while only four of them continued the measurements up to 12 months of follow-up [21,22,29,30]. Notably, the study conducted by Gerken et al. [21] observed a significant decrease in BCM at 6 months post-surgery, with participants having initial values of 43.78 ± 12.63 kg and 34.38 ± 9.11 kg at 6 months. Regarding longer-term follow-up, it was observed that after 12 months post-intervention, these values decreased to 33.81 ± 8.73 kg, indicating a more significant decrease in the first 6 months post-intervention.

**Figure 8:**
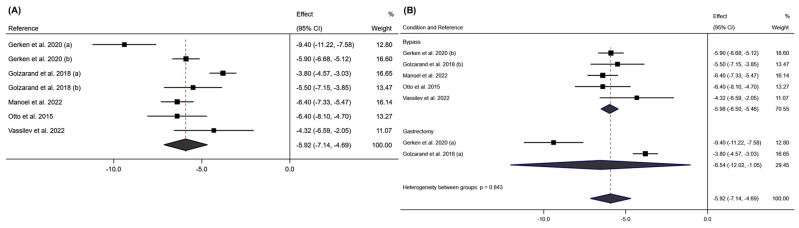
(**A**) Forest plot detailing the effect size and 95% confidence intervals (CI) for the effect of surgical intervention on body cell mass at 6 months post-intervention; (**B**) forest plot detailing the effect size and 95% confidence intervals (CI) for the effect of each type of surgical intervention on body cell mass at 6 months post-intervention. (a) sleeve gastrectomy; (b) Roux-en-Y gastric bypass [18,20,21,24,29].

The meta-analysis of the BCM variable showed that bariatric surgery significantly influences this variable, with an average effect size at six months post-surgery of −5.92; 95% CI: −7.14 to −4.69 (*p* < 0.001; I^2^ = 86.1%; 95% CI: 0.0% to 94.91%; *p* < 0.001). According to the surgical intervention performed, it was observed that the SG intervention reduced this variable more significantly than RYGB (SG: effect size: −6.54; 95% CI: −12.02 to −1.05 (*p* < 0.020; I^2^ = 98.8%; *p* < 0.001); RYGB: effect size: −5.98; 95% CI: −6.50 to −5.46 (*p* < 0.001; I^2^ = 0.0%; *p* = 0.487)). These results can be seen in Figure 8.

**Figure 9:**
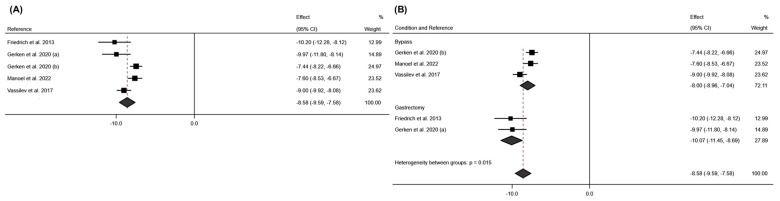
(**A**) Forest plot detailing the effect size and 95% confidence intervals (CI) for the effect of surgical intervention on body cell mass at 12 months post-intervention; (**B**) forest plot detailing the effect size and 95% confidence intervals (CI) for the effect of each type of surgical intervention on body cell mass at 12 months post-intervention. (a) sleeve gastrectomy; (b) Roux-en-Y gastric bypass [21,22,29,30].

In Figure 9, the meta-analysis for this variable at 12 months post-intervention can be seen. It shows that BCM had an average effect size of −8.58; 95% CI: −9.59 to −7.58 (*p* < 0.001; I^2^ = 73.9%; 95% CI: 0.0% to 91.3% *p* < 0.004), with the SG intervention showing a more significant reduction at 12 months post-intervention compared to RYGB (SG: Effect size: −10.07; 95% CI: −11.45 to −8.69 (*p* < 0.001; I^2^ = 0.0%; *p* = 0.871); RYGB: Effect size −8.00; 95% CI: −8.96 to −7.04 (*p* < 0.001; I^2^ = 72.3%; *p* < 0.027)). Regarding bias evaluation, the Doi plot and LFK index for this variable at 6- and 12-months post-intervention can be found in Figure S10 of the Supplementary Materials. In both cases, major asymmetry was observed.

In the sensitivity analysis for the BCM variable, no significant alterations in effect size were observed when excluding one-by-one studies. The overall effect of −5.92 (95% CI: −7.14 to −4.69) remained consistent, and variations were within a range of no more than 10% of the original effect size. The results of this analysis can be found in Figure S11 in the Supplementary Materials.

#### 3.4.4. Weight and BMI Changes

Studies conducted across various geographical locations, including Germany, Brazil, Iran, and Spain, collectively provide extensive data demonstrating significant reductions in both weight and BMI following bariatric procedures such as RYGB and SG [18,19,20,21,23,24,25,26,27,29,30].

A notable study by Golzarand et al. [24] from Iran on sleeve gastrectomy patients revealed a decrease in BMI from 39.5 kg/m^2^ to 30.3 kg/m^2^ within six months post-surgery, a pattern mirrored in most of the studies examined. This significant BMI reduction is paralleled by profound weight loss, exemplified in the same study, where the initial weight dropped from 101 kg to 78.4 kg in the same timeframe. Such trends were not isolated, as similar observations were made in studies from Brazil by Florencio et al. [19] and Venâncio et al. [23], Germany by Vassilev et al. [18,22] and Gerken et al. [21], and Spain by Martínez et al. [27] and Manoel et al. [29], among others.

**Figure 10:**
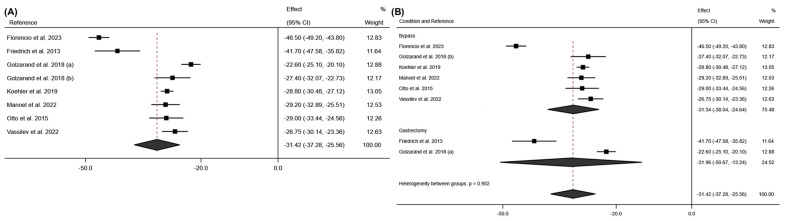
(**A**) Forest plot detailing the effect size and 95% confidence intervals (CI) for the effect of surgical intervention on weight at 6 months post-intervention; (**B**) Forest plot detailing the effect size and 95% confidence intervals (CI) for the effect of each type of surgical intervention on weight at 6 months post-intervention. (a) sleeve gastrectomy; (b) Roux-en-Y gastric bypass [18,19,20,24,25,29].

**Figure 11:**
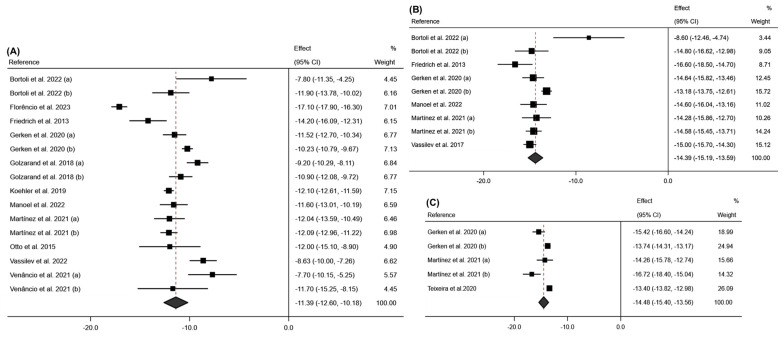
Forest plot detailing the effect size and 95% confidence intervals (CI) for the effect of surgical intervention on BMI at (**A**) 6 months post-intervention; (**B**) 12 months post-intervention; (**C**) 24 months post-intervention. (a) Sleeve Gastrectomy; (b) Roux-en-Y Gastric Bypass [18,19,20,21,22,23,24,25,26,27,28,29,30].

For the weight variable, a total of 7 studies measured weight variation at 6 months post-surgery [18,19,22,24,25,29,30]. A high mean effect was observed for this variable (effect size: −31.42; 95% CI: −37.28 to −25.26; *p* < 0.001) (I^2^ = 96.5%; 95% CI: 77.3% to 98.7%; *p* < 0.001). These results are reaffirmed by the measurement of BMI at 6 months, which also showed a large mean effect (effect size: −11.39; 95% CI: −12.60 to −10.18; *p* < 0.001) (I^2^ = 94.4%; 95% CI: 77.0% to 97.5% *p* < 0.001). These results can be seen in Figure 10 and Figure 11.

**Figure 12:**
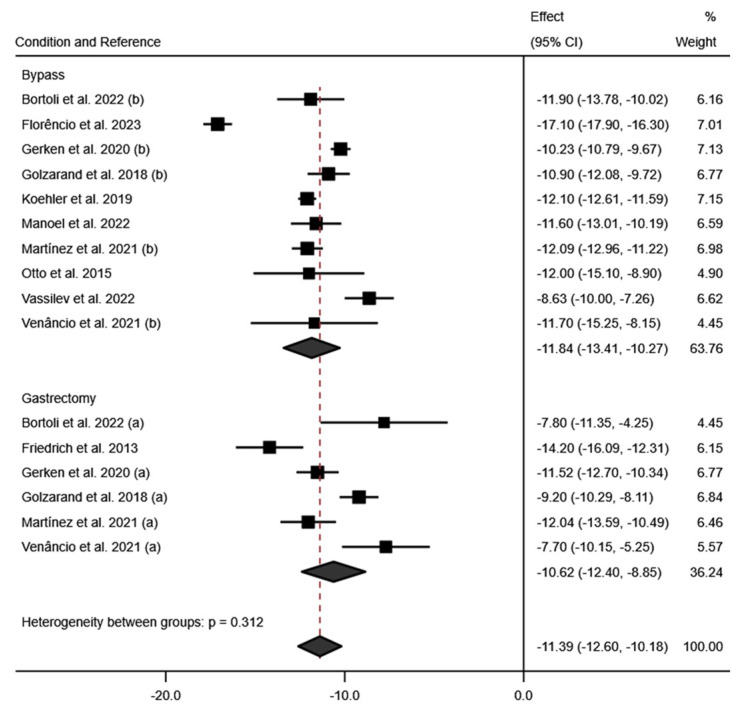
Forest plot detailing the effect size and 95% confidence intervals (CI) for the effect of each type of surgical intervention on BMI at 6 months post intervention. (a) sleeve gastrectomy; (b) Roux-en-Y gastric bypass [18,19,20,21,23,24,25,26,27,29,30].

According to the surgical technique employed, a greater reduction in BMI is observed at six months post-intervention with the RYGB technique (effect size: −11.84; 95% CI: −13.41 to −10.27; *p* < 0.001) (I^2^ = 95.5%; *p* < 0.001) compared to SG (effect size: −10.62; 95% CI: −12.40 to −8.85; *p* < 0.001) (I^2^ = 85.1%; *p* < 0.001). The data can be seen in Figure 12.

The data across these studies consistently showed a marked reduction in weight and BMI at six months post-operation, with some studies extending their follow-up to 12 and even 24 months, providing insight into the long-term sustainability of bariatric surgery benefits. Regarding the follow-up of the weight variable, there were not enough studies that monitored weight at 12 and 24 months. However, there were sufficient data to calculate the effect size for the BMI variable at 12 and 24 months of follow-up. For instance, the Teixeira et al. [28] study from Brazil reported ongoing reductions in BMI up to 24 months post-surgery, emphasizing the enduring impact of bariatric interventions. This reduction in BMI at 12 and 24 months of follow-up can be seen in Figure 11. Regarding the surgical technique employed, at 12 months post-intervention, a greater reduction in BMI was observed in patients who underwent the RYGB surgery (effect size: −14.36; 95% CI: −15.26 to −13.47; *p* < 0.001) (I^2^ = 78.4%; *p* < 0.001) compared to those who underwent the SG surgery (effect size: −14.15; 95% CI: −16.13 to −12.17; *p* < 0.001) (I^2^ = 78.1%; *p* < 0.003). However, at 24 months post-intervention, this trend reverses, with greater BMI loss in SG patients (effect size: −14.95; 95% CI: −16.06 to −13.83; *p* < 0.001) (I^2^ = 28.3%; *p* = 0.238) than in RYGB patients (Effect size: −14.23; 95% CI: −15.31 to −13.15; *p* < 0.001) (I^2^ = 86.0%; *p* < 0.001). These results can be seen in Figure S12 of the Supplementary Materials.

The Doi plots and LFK indices for the weight and BMI variables can be seen in Figure S13 of the Supplementary Material. For weight at 6 months post-intervention, major asymmetry was observed (LFK index = −2.12). For BMI, minor asymmetry was observed at 6 months post-intervention as well as at 12 and 24 months of follow-up (LFK index 6 months = −1.47; LFK index 12 months = −1.83; LFK index 24 months = −1.77).

Sensitivity analysis for these variables indicated no significant changes in the overall effect sizes. The data are presented in Figure S14 of the Supplementary Materials.

## 4. Discussion

To our knowledge, this is the first meta-analysis to comprehensively examine changes in PhA and other BIA measures following bariatric surgery, comparing the effects of SG and RYGB. The main findings of this study are as follows: (a) a significant decrease in PhA in the first 6 months post-surgery, with a sustained trend at 12 and 24 months; (b) a greater reduction in PhA in patients undergoing RYGB compared to SG in the short term, equalizing in the medium term and reversing in the long term; and (c) a notable reduction in FM and FFM in both types of surgery, with differences in the patterns of loss over time.

### 4.1. Phase Angle

Our meta-analysis included 13 studies that evaluated changes in PhA following bariatric surgery, with follow-ups at 6, 12, and 24 months. The results indicated a significant decrease in PhA in the first 6 months post-intervention, with an effect size of −1.00 (95% CI: −1.11 to −0.89; *p* < 0.001). This decline was consistent at 12 and 24 months, with effect sizes of −1.09 (95% CI: −1.15 to −1.02; *p* < 0.001) and −1.04 (95% CI: −1.17 to −0.92; *p* < 0.001), respectively. The heterogeneity among studies was considerable (I^2^ = 88.4% at 6 months, 49.8% at 12 months, and 85.0% at 24 months), suggesting significant variability in the reported results.

When comparing the two types of surgical intervention, we found that the reduction in PhA was greater in patients undergoing RYGB than in those with SG at 6 months (−1.02 vs. −0.96). However, at 12 months, the decrease in PhA was similar between both techniques, and at 24 months, SG patients showed a greater reduction in PhA compared to RYGB (−1.25 vs. −0.94).

Our study findings align with current literature, which also documents an initial decrease in PhA following bariatric surgery, primarily attributed to rapid body mass loss and changes in body composition [31,32]. This focus on PhA as a key indicator is supported by growing evidence of its utility over traditional nutritional markers like BMI or biochemical indices. Recent systematic reviews, such as those by Franco–Oliva et al. [33], Victoria–Montesinos et al. [34], and Arab et al. [35], suggest that PhA could be a more sensitive and specific marker of nutritional and health status in various clinical populations. For example, Victoria–Montesinos et al. highlighted that PhA is linked to improvements in handgrip strength and fat-free mass, indicating a parallel recovery in muscle function. Franco–Oliva et al. emphasized PhA’s strong correlations with inflammation markers like CRP while noting that it remains unaffected by acute physiological stress, making it a reliable marker of nutritional recovery. Additionally, Arab et al. discussed the inverse association between PhA and IL-6 levels, indicating its potential as a marker of systemic inflammation and catabolic states.

The greater reduction in PhA within the first 6 months post-RYGB may be due to the more invasive nature of this surgery compared to SG [36]. RYGB involves more extensive reconfiguration of the digestive system, resulting in more rapid and significant weight loss initially, but also greater loss of FFM and changes in body composition [37]. This intervention can also more significantly alter nutrient absorption, contributing to a more pronounced decrease in PhA due to changes in nutritional status and cellular quality [21].

At 12 months, the similar reduction in PhA between both groups may indicate stabilization in the rate of weight loss and body adjustments after the initial post-surgical phase. This suggests that regardless of the type of surgery, the body begins to metabolically adapt, leading to a similar reduction in PhA in the medium term [38]. The greater reduction in PhA observed at 24 months in SG patients compared to RYGB may be related to differences in long-term nutrient absorption and metabolic adaptations [39]. SG, although less invasive, could lead to more efficient long-term nutrient absorption compared to RYGB, resulting in better recovery of FFM and stabilization or even an increase in PhA [40]. This hypothesis is supported by studies showing greater stabilization of nutritional status in SG patients in the long term [41,42].

To address these reductions in PhA, several studies have identified specific interventions that effectively increase PhA in clinical populations. Campa et al. [43] showed through a systematic review and meta-analysis that resistance training in older adults significantly increased PhA, along with improvements in muscle mass and cellular health markers. Similarly, Di Renzo et al. [44] found that an immuno-enhanced nutritional formula with omega-3 fatty acids and arginine effectively increased PhA in cancer patients, suggesting enhanced cellular integrity and reduced inflammation. Furthermore, Basiri et al. [45] highlighted the benefits of a targeted high-protein nutritional intervention in overweight and obese diabetic patients with foot ulcers, which led to significant improvements in PhA and better muscle mass retention. Additionally, Rezazadegan et al. [46] examined the relationship between dietary inflammatory index and PhA, finding that dietary interventions reducing inflammatory load positively impacted PhA and overall cellular health. These findings indicate that implementing tailored nutritional and physical interventions in post-bariatric surgery patients could be crucial to counteracting the decline in PhA, supporting better muscle mass retention, cellular health, and overall recovery. Such approaches may optimize long-term outcomes for patients undergoing bariatric surgery, especially given the rapid body mass changes and nutrient absorption challenges inherent to these procedures.

Di Vincenzo et al. [11], in their systematic review, highlighted the importance of PhA as a marker of cellular and nutritional health in obese patients undergoing bariatric surgery. They found that PhA decreases significantly following bariatric surgery, with more substantial reductions observed in more invasive procedures like RYGB compared to SG. These reductions in PhA are closely linked to the loss of FFM and changes in muscle quality post-surgery. Di Vincenzo et al. [11] emphasized that monitoring PhA is crucial for identifying potential nutritional deficits and muscle mass losses that can compromise recovery and patient quality of life. Their review supports our findings of significant changes in body composition parameters post-surgery and underscores the necessity for comprehensive monitoring and tailored nutritional strategies to optimize long-term outcomes for bariatric surgery patients.

Additionally, the relationship between PhA and other clinical markers, such as IL-6 and CRP, underscores PhA’s potential to serve as an indicator of systemic inflammation and catabolism [47]. Future studies should further explore these relationships and establish protocols for using PhA in the post-surgical follow-up and management of obese patients.

### 4.2. Fat Mass, Body Cell Mass and Body Composition

At 6 months post-surgery, the reduction in FM was notable, with an effect size of −20.32 (95% CI: −22.86 to −17.77; *p* < 0.001), indicating significant FM loss. This effect intensified at 12 months, with an effect size of −27.58 (95% CI: −32.58 to −22.57; *p* < 0.001), suggesting that FM loss continues and intensifies in the first year post-surgery. However, at 24 months, the available information was insufficient for a detailed analysis of FM, highlighting the need for more long-term longitudinal studies.

FFM also showed a significant reduction. At 6 months, the effect size was −8.28 (95% CI: −10.76 to −5.80; *p* < 0.001), and at 12 months, the decrease was even greater with an effect size of −10.51 (95% CI: −12.81 to −8.94; *p* < 0.001). These reductions reflect the loss of muscle mass and other lean tissues, a critical consideration in evaluating the success and potential complications of bariatric surgery.

When comparing surgical techniques, it was observed that RYGB resulted in a greater reduction of FM and FFM at 6 months compared to SG. The effect size for FM was −21.06 (95% CI: −23.71 to −18.41) for RYGB and −18.32 (95% CI: −22.42 to −14.21) for SG. This trend reversed at 12 months, where FM reduction was greater in patients undergoing SG. These findings indicate that although both techniques are effective for FM loss, the patterns of body composition change may differ between procedures [48].

The greater reduction in FM and FFM at 6 months post-RYGB can be explained by the greater magnitude of digestive alterations and malabsorption that this procedure induces compared to SG. RYGB, which, by involving the creation of a small gastric pouch and bypassing the duodenum, significantly reduces nutrient absorption, leading to rapid loss of both fat and lean mass [37,49]. Additionally, the reduction in FFM may reflect the loss of muscle mass due to acute malnutrition and protein catabolism during the initial weight loss phase [50].

At 12 months, the reverse pattern observed, with greater FM reduction in SG patients, could be due to better muscle mass preservation in this group, allowing for greater mobilization of fat reserves as the body adapts to the sustained caloric deficit. This suggests that SG, by better preserving digestive function and nutrient absorption, could facilitate a more balanced metabolic adjustment in the long term [51].

Hormonal changes also play a crucial role in regulating fat mass and body composition following bariatric surgery. The reduction of ghrelin, the hormone that stimulates appetite, is more pronounced after RYGB due to the resection of the gastric fundus, where this hormone is produced [52]. This decrease contributes to a greater reduction in caloric intake and, consequently, more rapid weight loss.

On the other hand, the increase in anorexigenic hormones such as leptin and peptide YY (PYY) after bariatric surgery also helps reduce appetite and increase satiety, facilitating the maintenance of weight loss in the long term [52,53].

The greater reduction in FM and FFM observed in RYGB may be related to the hormonal changes mentioned. RYGB tends to cause a greater reduction in ghrelin levels and a greater increase in PYY levels compared to SG, which may explain the greater loss of fat and lean mass in the short term [37,54]. However, better preservation of digestive function and nutrient absorption in SG could allow for better metabolic adaptation and muscle mass recovery in the long term, as seen in changes in FFM at 12 and 24 months [54].

Our findings are consistent with existing literature documenting significant improvements in body composition following bariatric surgery. Reduction in FM and improvement in LBM are key indicators of success in obesity intervention [55]. The literature suggests that these improvements in body composition not only contribute to weight loss but also have important implications for metabolic health and the reduction of comorbidities associated with obesity [56].

In alignment with our results, Nuijten et al. revealed that postbariatric loss of muscle tissue could negatively affect long-term health due to its role in various bodily processes, such as metabolism and functional capacity [50]. This meta-analysis aimed to unravel time-dependent changes in the magnitude and progress of LBM, FFM, and skeletal muscle mass (SMM) loss following bariatric surgery. At 12-month postsurgery, pooled LBM loss was 8.13 kg [95% CI 7.26; 9.01]. FFM loss and SMM loss were 8.23 kg [95% CI 5.73; 10.74] and 3.18 kg [95% CI 0.71; 5.64], respectively. About 55% of 12-month LBM loss occurred within 3-month postsurgery, followed by a more gradual decrease up to 12 months. Similar patterns were seen for FFM and SMM. These findings support our observation of significant reductions in both FM and FFM at 6- and 12-months post-surgery, highlighting the critical need for interventions to mitigate muscle mass loss perioperatively.

Furthermore, the evaluation of BCM post-surgery provides additional insights into the changes in metabolically active tissue and potential nutritional deficits that can impact recovery and patient quality of life. Our results showed a significant decrease in BCM at 6 months post-surgery, with a meta-analysis showing an average effect size at six months post-surgery of −5.92; 95% CI: −7.14 to −4.69 (*p* < 0.001; I^2^ = 86.1%; *p* < 0.001). At 12 months, the effect size was −8.58; 95% CI: −9.59 to −7.58 (*p* < 0.001; I^2^ = 73.9%; *p* < 0.004). Pipek et al. [57] highlight similar findings, demonstrating that bariatric surgery, particularly RYGB and SG, significantly impacts various body composition parameters, including muscle mass. Their meta-analysis reported substantial muscle mass loss, emphasizing the importance of monitoring BCM to ensure patients maintain a healthy cell mass. This is crucial for basal metabolism and physical functionality, thus influencing the long-term outcomes of bariatric surgery. This underscores the need for comprehensive post-surgical follow-up and nutritional strategies to mitigate these losses and support patient recovery.

### 4.3. Weight and BMI

At 6 months post-surgery, the analysis revealed an average weight reduction of −31.42 (95% CI: −37.28 to −25.26; *p* < 0.001), with considerable heterogeneity (I^2^ = 96.5%; *p* < 0.001). This decrease is a clear indicator of the initial success of bariatric surgery in reducing body weight. The analysis of BMI showed a significant reduction at 6 months post-surgery, with an effect size of −11.39 (95% CI: −12.60 to −10.18; *p* < 0.001) and considerable heterogeneity (I^2^ = 94.4%; *p* < 0.001). This trend continued at 12- and 24-months post-surgery, with effect sizes of −14.36 (95% CI: −15.26 to −13.47; *p* < 0.001) and −14.23 (95% CI: −15.31 to −13.15; *p* < 0.001), respectively, indicating sustained reduction in BMI in the long term.

When comparing surgical techniques, a greater reduction in BMI was observed in patients undergoing RYGB at 6 months, with an effect size of −11.84 (95% CI: −13.41 to −10.27; *p* < 0.001) compared to −10.62 (95% CI: −12.40 to −8.85; *p* < 0.001) in SG patients. At 12 months, this trend continued, although the differences between the two techniques attenuated. At 24 months, SG patients showed a greater reduction in BMI than those with RYGB, suggesting that long-term effects may vary according to the type of intervention.

As mentioned in the section on FM, BCM, and body composition, the greater reduction in weight and BMI at 6 months post-RYGB can be explained by the combined effects of restriction and malabsorption characteristic of this procedure [40]. Additionally, hormonal changes induced by RYGB, such as the greater reduction in ghrelin levels and the increase in PYY and GLP-1 levels, contribute to greater satiety and reduced caloric intake [53].

At 12 months, the BMI reduction remains similar between both techniques, indicating that, in the medium term, the impact of hormonal and metabolic changes induced by both surgeries tends to balance out. SG, although less invasive, also induces significant changes in appetite-regulating hormones, such as ghrelin, and improves insulin sensitivity, favoring sustained weight loss. For example, one study found that ghrelin reduction is significant in both SG and RYGB, but the duration of suppression may be longer in RYGB, explaining the initial differences in weight loss [58].

Another possible explanation for the difference in long-term results between SG and RYGB could be related to individual responses to surgery and differences in adherence to post-surgical dietary recommendations [59]. Studies have shown that SG patients may have better adherence to dietary recommendations due to lower technical complexity and fewer postoperative complications [60]. On the other hand, RYGB patients may experience more difficulties with nutrient absorption, affecting the sustainability of long-term weight loss [37].

Our findings are consistent with existing literature documenting significant reductions in weight and BMI following bariatric surgery. The rapid and sustained reduction in weight and BMI is one of the most documented benefits of bariatric surgery [61]. Pipek et al. [57] corroborate our findings, showing that bariatric surgery results in superior long-term outcomes compared to pharmacological treatments. The meta-analysis revealed that surgical procedures were more effective for weight loss with a mean difference of −22.05 kg (95% CI: −28.86; −15.23) and resulted in significant improvements in cardiovascular and metabolic parameters, such as total cholesterol, triglycerides, and HbA1c levels. Pipek et al. also emphasized that the choice of surgical technique influences long-term outcomes, with RYGB showing a greater initial reduction in BMI and weight, like our observations [57]. However, their findings also highlight the importance of patient adherence to post-surgical dietary recommendations and the role of hormonal changes, which align with our conclusions regarding the differential effects of RYGB and SG on weight loss sustainability.

### 4.4. Limitations

The present meta-analysis has several limitations that need to be acknowledged. First, the considerable heterogeneity observed among the included studies is reflected in the different effect sizes and variability in results, especially in PhA parameters, weight, BMI, and body composition. Several reasons contribute to this heterogeneity, including differences in study designs, study populations, surgical procedures, follow-up protocols, and measurement methodologies.

Additionally, although this meta-analysis focused on two main surgical procedures, RYGB and SG, specific techniques and variations in surgical practice among centers and surgeons may influence the outcomes. For example, the extent of gastric resection in SG or the length of the alimentary limb in RYGB can vary, which may affect the efficacy of weight loss and changes in body composition [62].

Moreover, the methodologies used to measure bioimpedance parameters, weight, BMI, and body composition differ among studies. The accuracy and reproducibility of BIA measurements can vary depending on the devices used, measurement conditions, and standardization of procedures. The lack of standardization in measurements can introduce bias and variability in results, affecting the overall interpretation of the meta-analysis.

The risk of bias is another important limitation of this meta-analysis. Although the ROBINS-I tool was used to assess the risk of bias in non-randomized studies, most of the included studies presented a moderate to high risk of bias, mainly due to residual confounding and participant selection.

Another significant limitation is the lack of long-term data. While several studies report outcomes at 6 and 12 months, there is a scarcity of studies providing data at 24 months or more. This limits our ability to evaluate the sustained effects of bariatric surgery on body composition, PhA, weight, and BMI.

## 5. Conclusions

In conclusion, this meta-analysis provides robust evidence on the effects of bariatric surgery on bioimpedance parameters, including PhA, weight, BMI, and body composition in individuals with obesity. The findings suggest a significant decrease in PhA in the first 6 months post-surgery, with a trend towards stabilization or improvement in the long term in some patients, highlighting the physiological and metabolic restructuring that accompanies surgically induced weight loss.

To improve the clinical management of patients with obesity, it is essential to continue investigating the long-term effects of bariatric surgery, standardize measurement and follow-up protocols, and further explore the differences between surgical techniques. Additionally, monitoring parameters such as PhA can provide a more comprehensive view of changes in body composition and cellular health, helping to optimize treatment strategies and post-surgical follow-up.

## Figures and Tables

**Table 1:** Search strategy.

Field	Description
Databases	MEDLINE (via PubMed), Scopus, Cochrane Library, Web of Science (WoS)
Search Date	Up to 2 May 2024
Search Terms	(“bariatric surgery” OR “bariatric patients” OR “weight loss surgery” OR “gastric bypass” OR “Roux-en-Y gastric bypass” OR “RYGB” OR “sleeve gastrectomy” OR “gastric banding” OR “obesity surgery” OR “duodenal switch” OR “biliopancreatic diversion”) AND (“bioimpedance” OR “bioimpedance analysis” OR “bioelectrical impedance” OR “bioelectrical impedance analysis” OR “BIA”) AND (“phase angle” OR “phase angle measurement” OR “PA”)

**Table 2:** Descriptive data of the study participants.

Reference	Country	Mean Age	N	Condition	BMI Initial	PhA Initial	Duration	Variables Collected	Phase Angle as Predictor of Different Outcomes
Vassilev et al. [18]	Germany	41.9	190	Obese (GB+SG)	42.96	6.38	6 months	BMI, Weight, FFM, FM, PhA	The study demonstrated a significant decrease in SMI values after RYGB, highlighting the correlation between SMI values and several body composition parameters such as body weight, phase angle, and body cell mass.
Florêncio et al. [19]	Brasil	38.5	69	Obese (GB)	44.2	4.6	24 months	BMI, Weight, FFM, FM, PhA, Handgrip Strength, Gait Speed, Appendicular Lean Mass (ALM) assessed using Dual Energy X-ray Absorptiometry (DXA)	The phase angle was notably lower than reference values, indicating significant alterations in cell membrane integrity even before the operation and further reduction after substantial weight loss. The research highlighted the phase angle’s correlation with sarcopenia, particularly regarding the decrease in muscle function. It was found that 17.86% of women and 9.09% of men in the preoperative period and 22.22% of women and 16.66% of men in the postoperative period exhibited low handgrip strength, a primary parameter for diagnosing sarcopenia.
Otto et al. [20]	Germany	42	18	Obese (GB)	43	6.5	6 months	BMI, Weight, FFM, FM, PhA	The phase angle, which reflects the quality of the lean body mass, showed a continuous and significant reduction from an initial 6.5 ± 0.8° to 5.4 ± 0.9° six months post-operation, indicating changes in cellular health and body composition.
Gerken et al. [21]	Germany	41.815	198	Obese (GB+SG)	50.105	6.205	24 months	BMI, weight, FFM, FM, PhA, and handgrip strength	A significant correlation was observed between preoperative phase angle and total weight loss up to 3 months after SG and up to 12 months after GB. The optimum cutoff for predicting a response of less than 50% excess weight loss was identified as a preoperative PhA of 6.0°.
Vassilev et al. [22]	Germany	-	173	Obese (GB)	48.3	6.4	24 months	Body weight, Body composition (Extracellular Mass, Body Cell Mass, (BCM), Lean Body Mass, Body Fat, Total Body Water) and PhA	A preoperative phase angle of 3.9° showed a sensitivity of 81% and a specificity of 54% for predicting the success of bariatric surgery, defined as less than 50% excess weight loss in 1 year.
Venâncio et al. [23]	Brasil	42.05	39	Obese (GB+SG)	41.8	6.95	6 months	BMI, weight, FFM, FM, PhA, serum total protein, transthyretin, C-reactive protein, serum thiobarbituric acid reactive substances (TBARS), advanced oxidation protein products (AOPP), and serum lipids.	RYGB might be more effective in attenuating oxidative damage after 6 months, with reductions in BMI suggesting a concurrent decrease in lipid oxidative damage. In the SG group, the changes in body impedance analysis parameters were inversely correlated with protein oxidative damage, suggesting different mechanisms of impact on oxidative stress between the two types of bariatric surgery.
Golzarand et al. [24]	Iran	40.45	43	Obese (GB+SG)	42.7	5.45	24 months	BMI, Weight, FFM, FM, PhA, Dietary intake (energy, protein, carbohydrate, fat, and fiber intake), Substrate oxidation (carbohydrate, protein, fat oxidation, and respiratory quotient), Regional body composition (trunk, limbs)	The decrease in phase angle postoperatively reflects a reduction in lean body mass or an increase in fat mass, highlighting its role as a potential marker for monitoring changes in nutritional status and body composition following bariatric surgery.
Koehler et al. [25]	Brasil	40.2	20	Obese (GB)	42.9	7.1	6 months	BMI, Weight, FFM, FM, PhA, serum C-reactive protein, alpha-1-acid glycoprotein, albumin, and transthyretin (TTR) concentrations	The study found that PhA was not associated with the Prognostic Inflammatory and Nutritional Index, although lower values of PhA were significantly correlated with lower serum transthyretin concentrations, suggesting that a reduction in PhA is associated with an increased nutritional risk.
Bortoli et al. [26]	Brasil	42.7	36	Obese (GB+SG)	41.8	7	12 months	BMI, Weight, FFM, FM, PhA, serum transthyretin (TTR), albumin, C-reactive protein, alpha-1-acid glycoprotein, and prognostic inflammatory and nutritional indices	PhA and serum TTR significantly decreased after both RYGB and SG, persisting throughout the follow-up period. There was a significant positive correlation between PhA and TTR in both RYGB and SG groups. These results suggest that a decrease in PhA at one-year follow-up after bariatric surgery might indicate a concomitant loss of visceral protein and a worsening of protein nutritional status.
Martínez et al. [27]	Spain	45.54	84	Obese (GB+SG)	44.16	6.875	24 months	BMI, Weight, FFM, FM, PhA, Basal Metabolism Rate (BMR), and Protein Metabolism (Transthyretin levels)	Significant findings include a continuous and substantial loss of FFM after bariatric surgery, amounting to about 21.71% of total weight loss 24 months post-surgery, regardless of the type of surgery (GB or SG) or protein metabolism changes. Phase angle, indicating cellular health, also significantly decreased post-surgery. Pre-surgery FFM and insulin resistance levels were predictive of FFM at 24 months, suggesting that the reduction in muscle mass post-surgery is influenced by pre-surgery conditions rather than surgery type or protein metabolism.
Teixeira et al. [28]	Brasil	39.1	379	Obese (GB) NAFLD	45.2	5.93	24 months	BMI, Weight, PhA	After bariatric surgery, there was a significant reduction in phase angle, indicating changes in body composition and cellular integrity. This reduction was directly correlated with weight loss but was not correlated with the improvement of NAFLD, showcasing the phase angle’s potential as a marker for assessing body composition and nutritional status changes post-surgery, despite the marked improvement in NAFLD.
Friedrich et al. [30]	Germany	45.4	27	Obese (SG)	51.7	5.5	12 months	BMI, Weight, FM LBM, BCM, PhA	The study found that phase angle, which decreased significantly in the LSG group, was associated with nutritional status and muscle mass. A lower phase angle indicated protein-energy malnutrition and muscle mass loss. Phase angle was used to predict changes in body composition and nutritional status post-surgery, showing significant associations with reductions in fat mass and increases in lean body mass.
Manoel et al. [29]	Brasil	40.0	21	Obese (GB)	43.3	7.0	12 months	BMI, Weight, FFM, FM, BCM, PhA	Physical activity may attenuate the PhA reduction that occurs after BS, with this parameter reflecting the engagement of these patients in this type of activity.

AOPP: advanced oxidation protein products; BCM: body cell mass; BMR: basal metabolism rate; BMI: body mass index; DXA: dual energy X-ray absorptiometry; FFM: fat-free mass; FM: fat mass; GB: gastric bypass; LBM: lean body mass; NAFLD: non-alcoholic fatty liver disease; PhA: phase angle; RYGB: Roux-en-Y gastric bypass; SG: sleeve gastrectomy; SMI: skeletal muscle index; TBARS: thiobarbituric acid reactive substances; TTR: transthyretin.

## Data Availability

Data are contained within the article.

## Supplementary Materials

The following supporting information can be downloaded at: https://www.mdpi.com/article/10.3390/jcm13226784/s1, Figure S1: Doi plot and Luis Furuya-Kanamori index determining the publication bias of the studies examined for the variable phase angle. LFK: Luis Furuya-Kanamori, (**A**) 6 months post-intervention, (**B**) 12 months post-intervention, (**C**) 24 months post-intervention; Figure S2: Leave-one-out sensitivity analysis for the variable phase angle. Figure S3: Forest plot detailing the effect size and 95% confidence intervals (CI) for the effect of each type of surgical intervention on phase angle at (**A**) 12 months post-intervention, (**B**) 24 months post-intervention. (a) sleeve gastrectomy; (b) Roux-en-Y gastric bypass; Figure S4: Forest plot detailing the effect size and 95% confidence intervals (CI) for the effect of each type of surgical intervention on fat mass at (**A**) 6 months post-intervention, (**B**) 12 months post-intervention. (a) sleeve gastrectomy, (b) Roux-en-Y gastric bypass; Figure S5: Forest plot detailing the effect size and 95% confidence intervals (CI) for the effect of each type of surgical intervention on free fat mass at (**A**) 6 months post-intervention, (**B**) 12 months post-intervention. (a) sleeve gastrectomy, (b) Roux-en-Y gastric bypass; Figure S6: Doi plot and Luis Furuya-Kanamori index determining the publication bias of the studies examined for: (**A**) Fat mass at 6 months post-intervention, (**B**) fat mass at 12 months post-intervention, (**C**) free fat mass at 6 months post-intervention, (**D**) free fat mass at 12 months post-intervention. LFK: Luis Furuya-Kanamori; Figure S7: forest plot detailing the effect size and 95% confidence intervals (CI) for the effect of each type of surgical intervention on lean body mass at (**A**) 6 months post-intervention, (**B**) 12 months post-intervention. (a) sleeve gastrectomy, (b) Roux-en-Y gastric bypass; Figure S8: Doi plot and Luis Furuya-Kanamori index determining the publication bias of the studies examined for lean body mass: (**A**) at 6 months post-intervention, (**B**) at 12 months post-intervention. LFK: Luis Furuya-Kanamori; Figure S9: Leave-one-out sensitivity analysis for the body composition variables: (**A**) Fat Mass, (**B**) fat free mass, (**C**) lean body mass; Figure S10: Doi plot and Luis Furuya-Kanamori index determining the publication bias of the studies examined for Body Cell Mass: (**A**) at 6 months post-intervention, (**B**) at 12 months post-intervention. LFK: Luis Furuya-Kanamori; Figure S11: Leave-one-out sensitivity analysis for the variable body cell mass; Figure S12: forest plot detailing the effect size and 95% confidence intervals (CI) for the effect of each type of surgical intervention on BMI at (**A**) 12 months post-intervention, (**B**) 24 months post-intervention. (a) sleeve gastrectomy, (b) Roux-en-Y gastric bypass; Figure S13: Doi plot and Luis Furuya-Kanamori index determining the publication bias of the studies examined for: (**A**) Weight at 6 months post-intervention, (**B**) BMI at 6 months post-intervention, (**C**) BMI at 12 months post-intervention, and (**D**) BMI at 24 months post-intervention. BMI: body mass index, LFK: Luis Furuya-Kanamori; Figure S14: leave-one-out sensitivity analysis for (**A**) weight and (**B**) body mass index; Figure S15: Prisma checklist.
