# Supplementary figures and images for: Comparative Effects of Sleeve Gastrectomy vs. Roux-en-Y Gastric Bypass on Phase Angle and Bioelectrical Impedance Analysis Measures: A Systematic Review and Meta-Analysis
Source: J Clin Med. 2024 Nov 11;13(22):6784. doi: 10.3390/jcm13226784 (PMC11594950; doi:10.3390/jcm13226784)

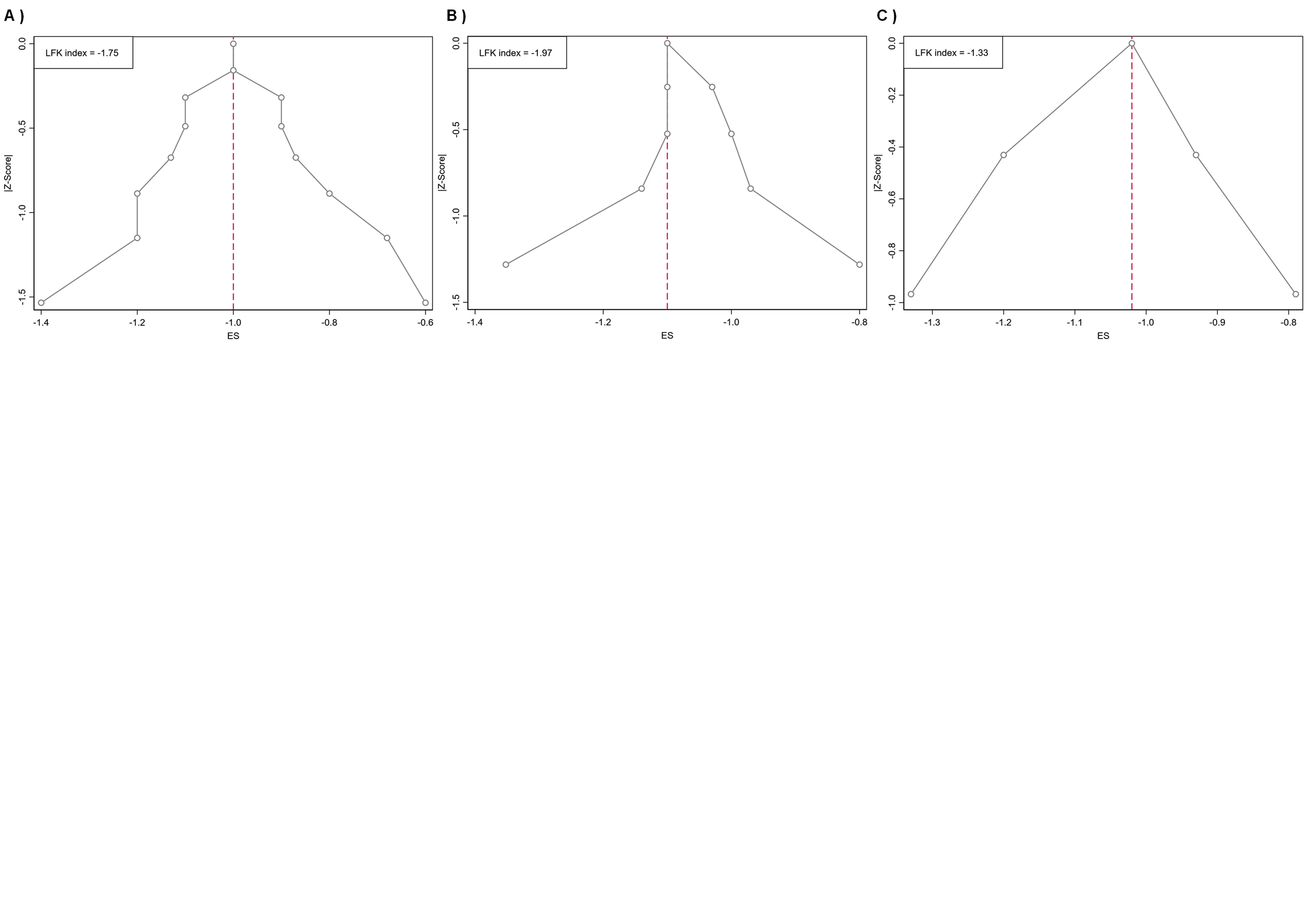

Supplement: Supplementary file 1 [file jcm-13-06784-s001.zip › Figure S1.tif]

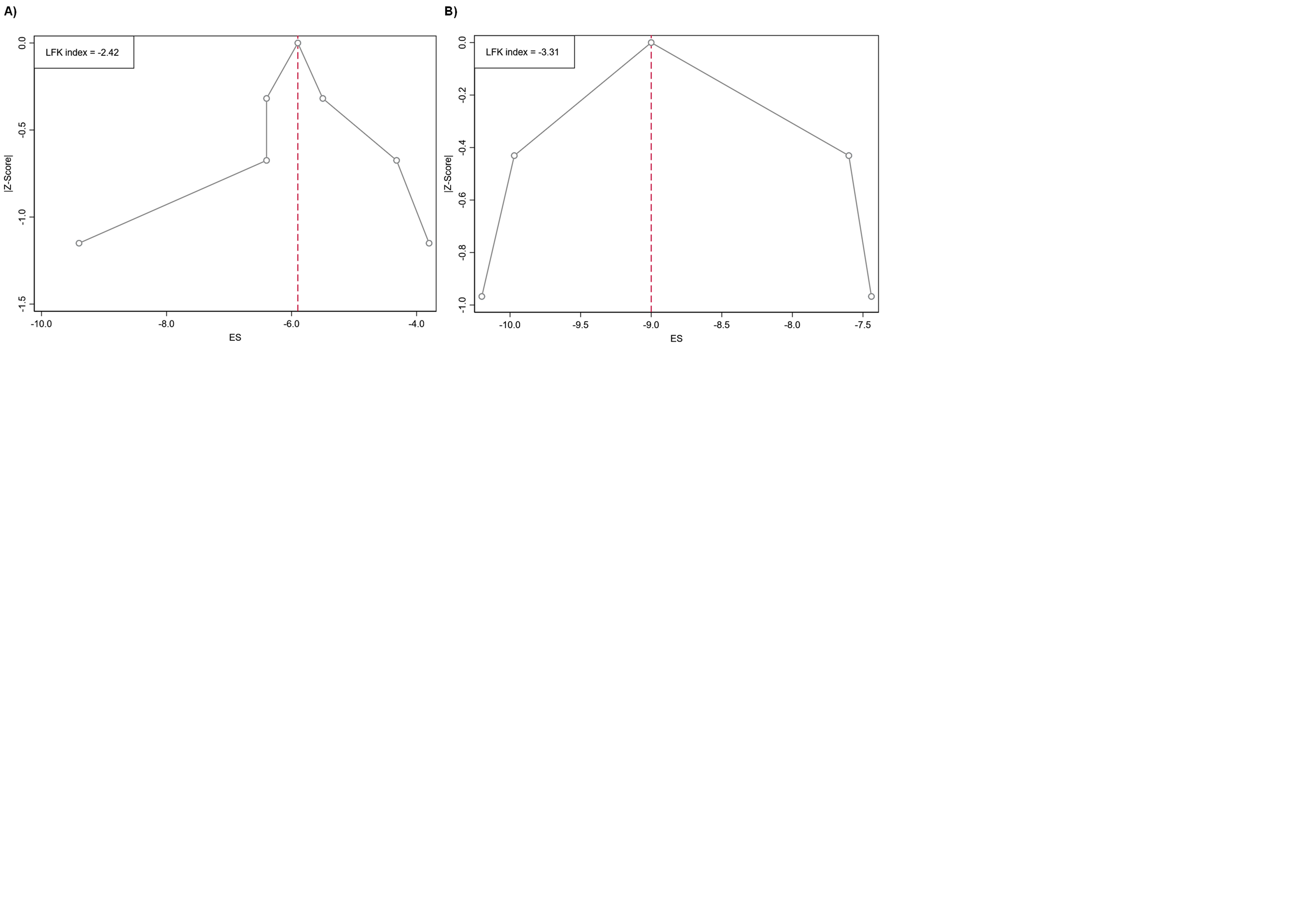

Supplement: Supplementary file 1 [file jcm-13-06784-s001.zip › Figure S10.tif]

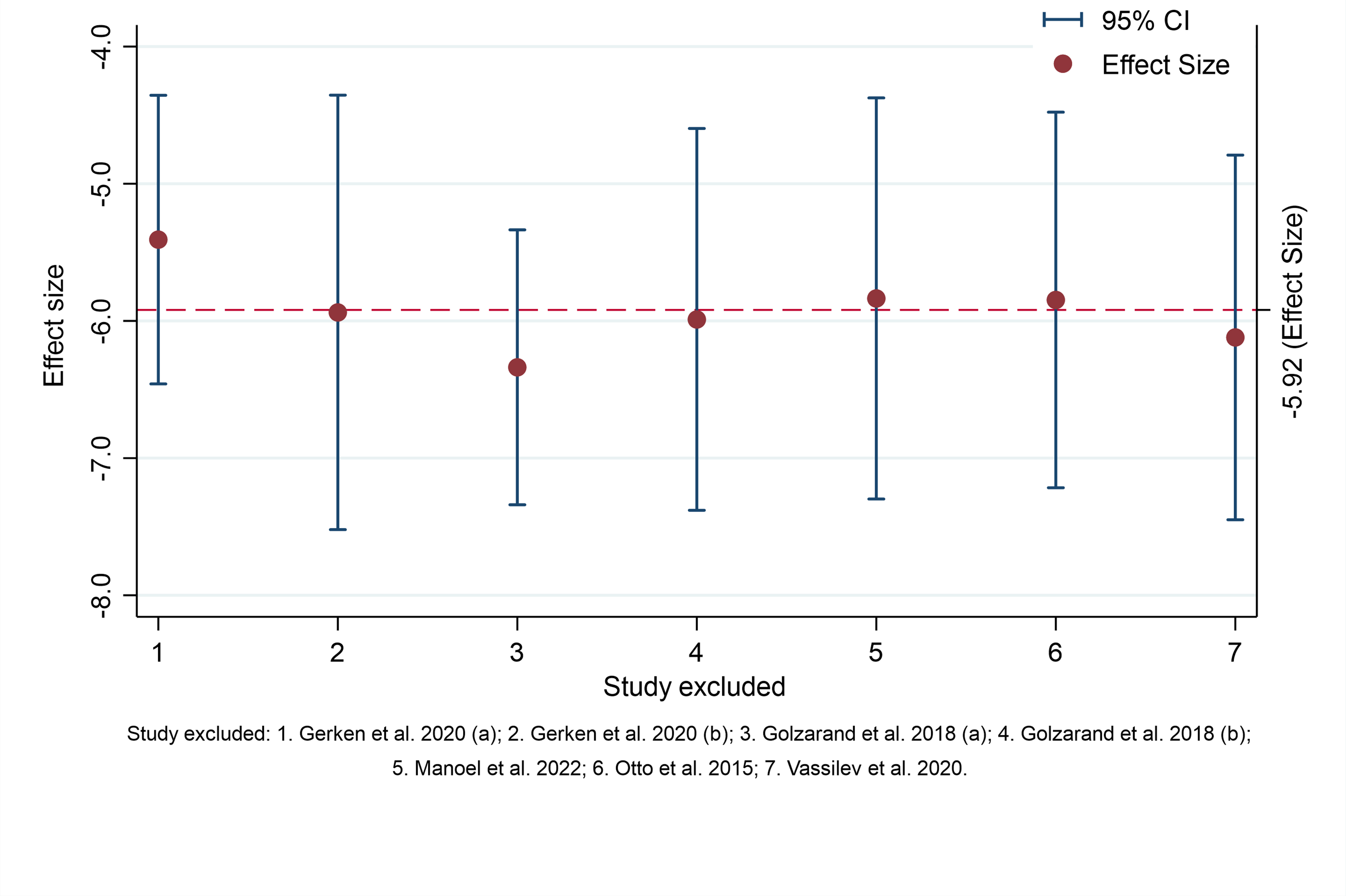

Supplement: Supplementary file 1 [file jcm-13-06784-s001.zip › Figure S11.tif]

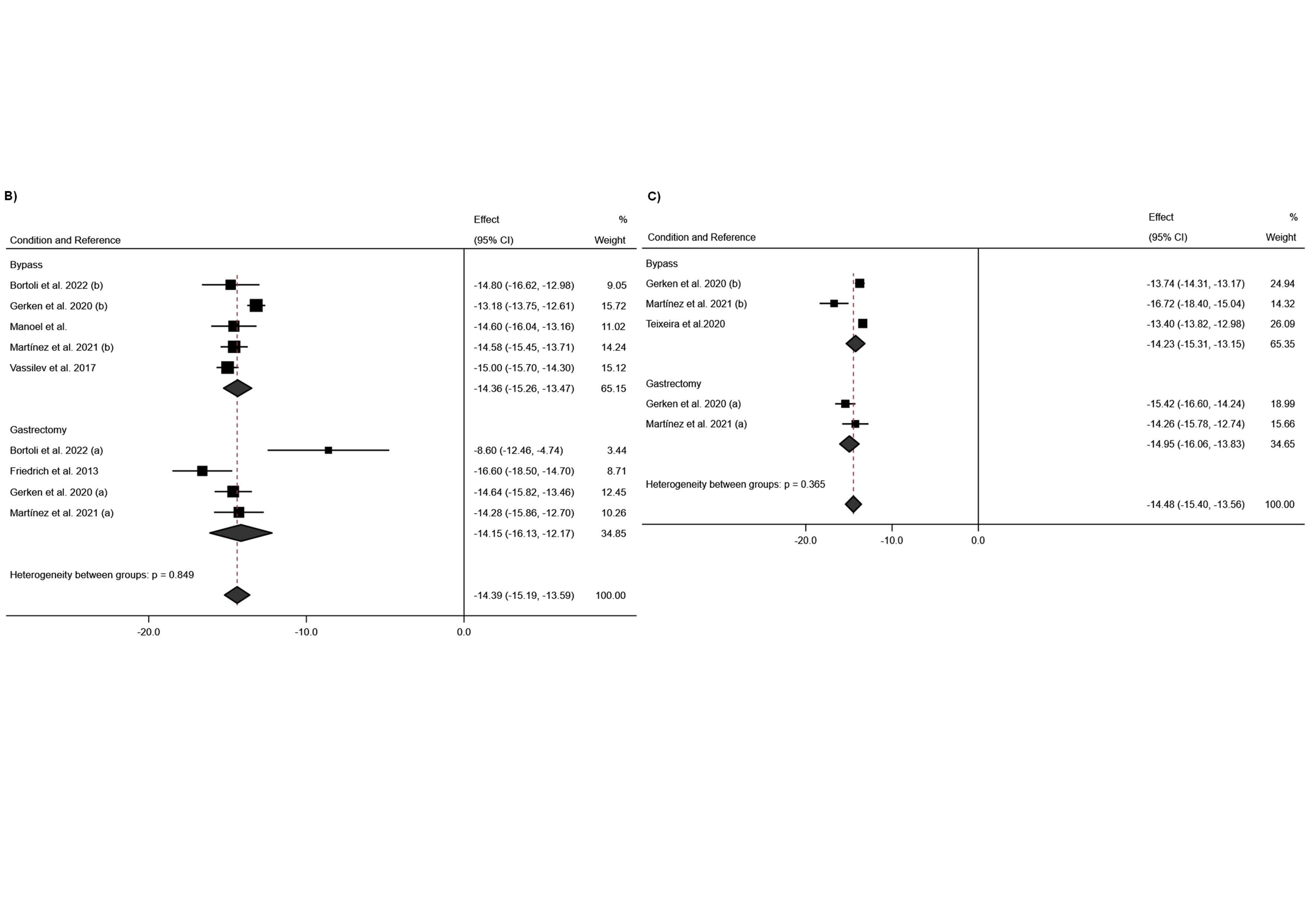

Supplement: Supplementary file 1 [file jcm-13-06784-s001.zip › Figure S12.tif]

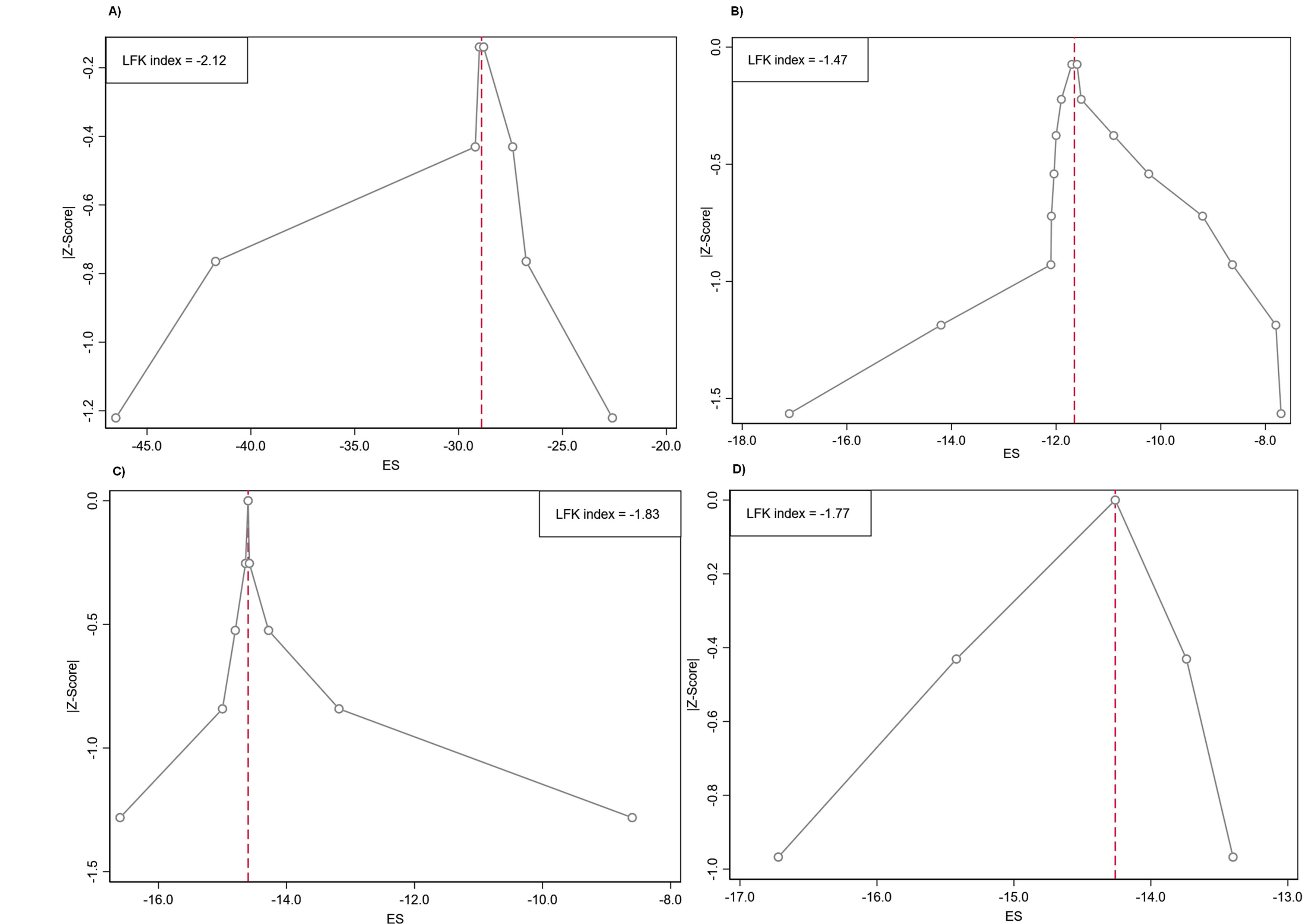

Supplement: Supplementary file 1 [file jcm-13-06784-s001.zip › Figure S13.tif]

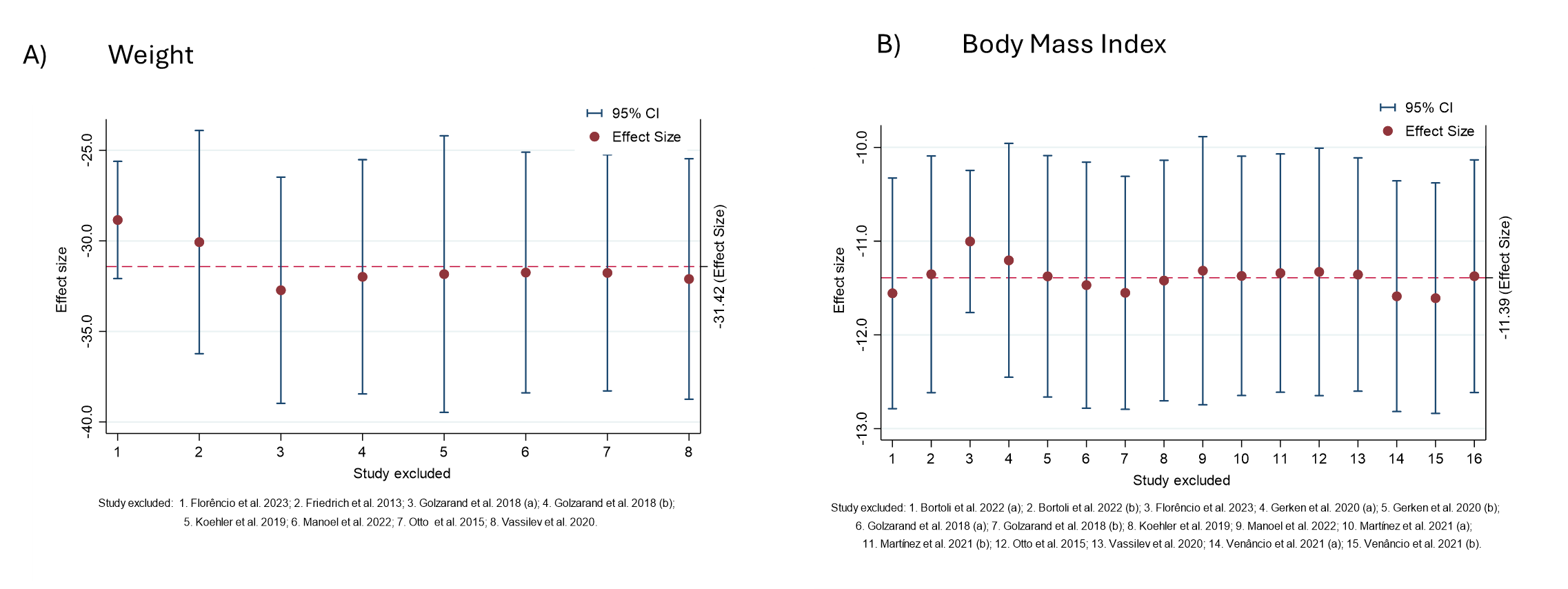

Supplement: Supplementary file 1 [file jcm-13-06784-s001.zip › Figure S14.tif]

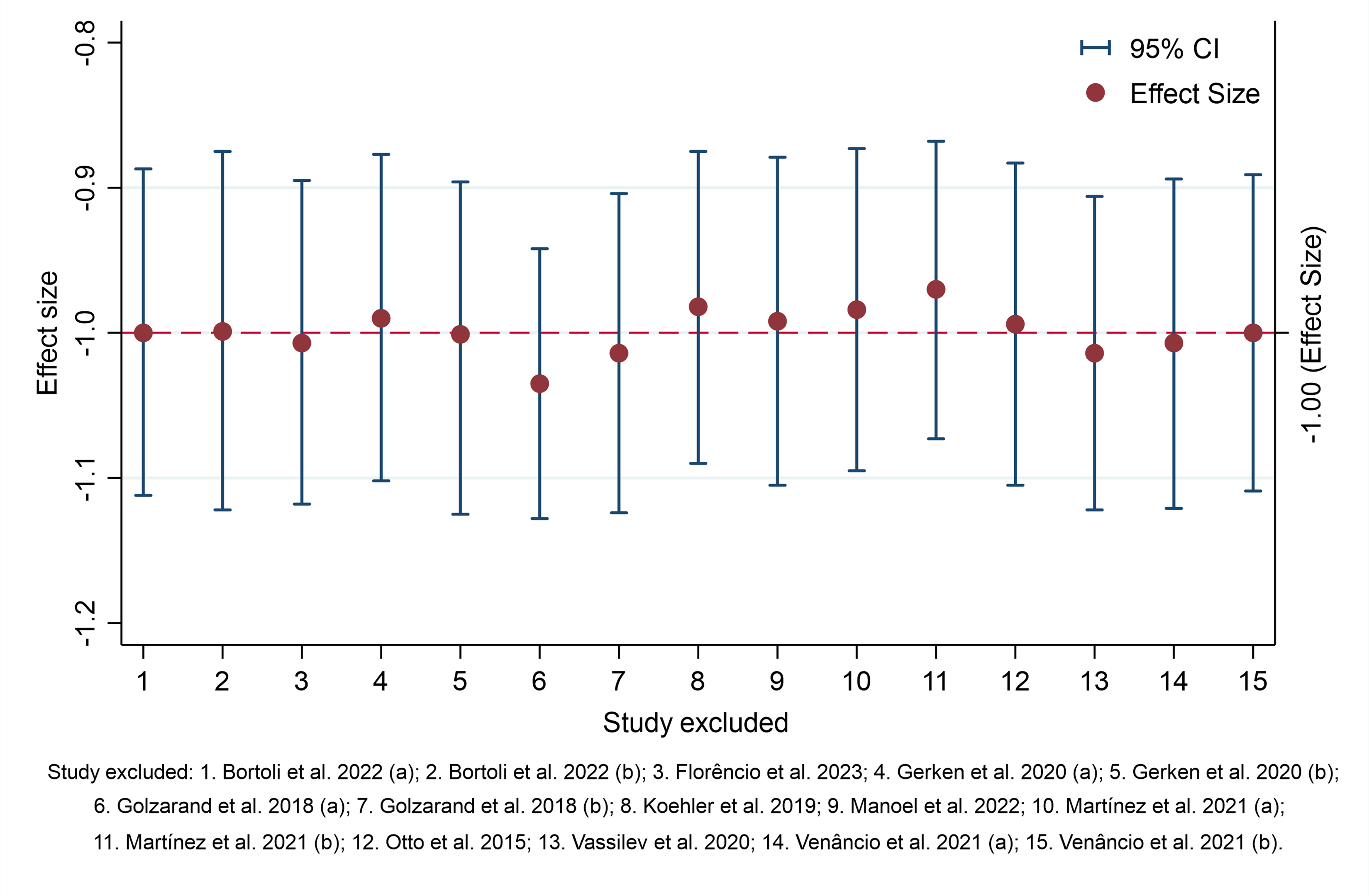

Supplement: Supplementary file 1 [file jcm-13-06784-s001.zip › Figure S2.tif]

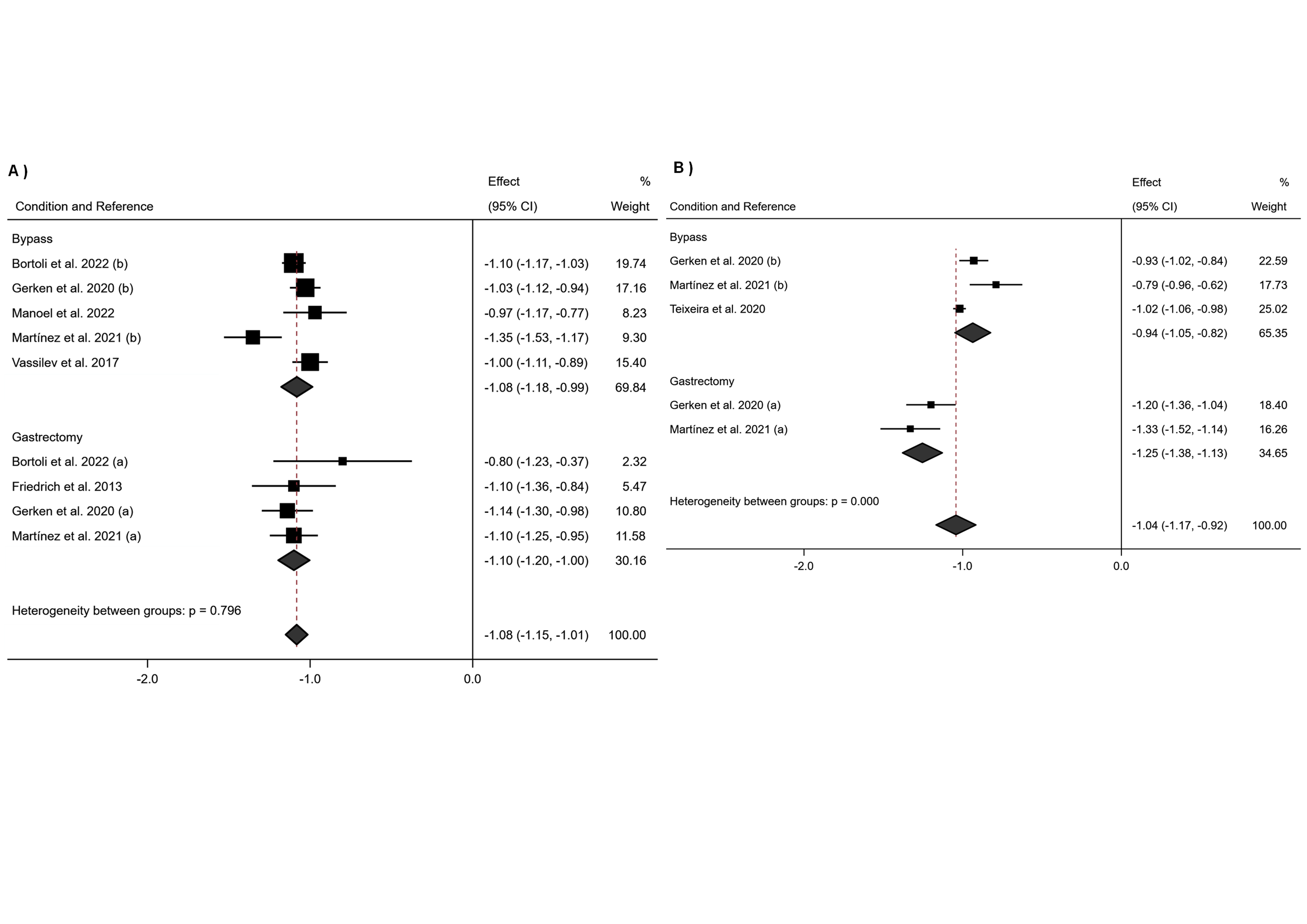

Supplement: Supplementary file 1 [file jcm-13-06784-s001.zip › Figure S3.tif]

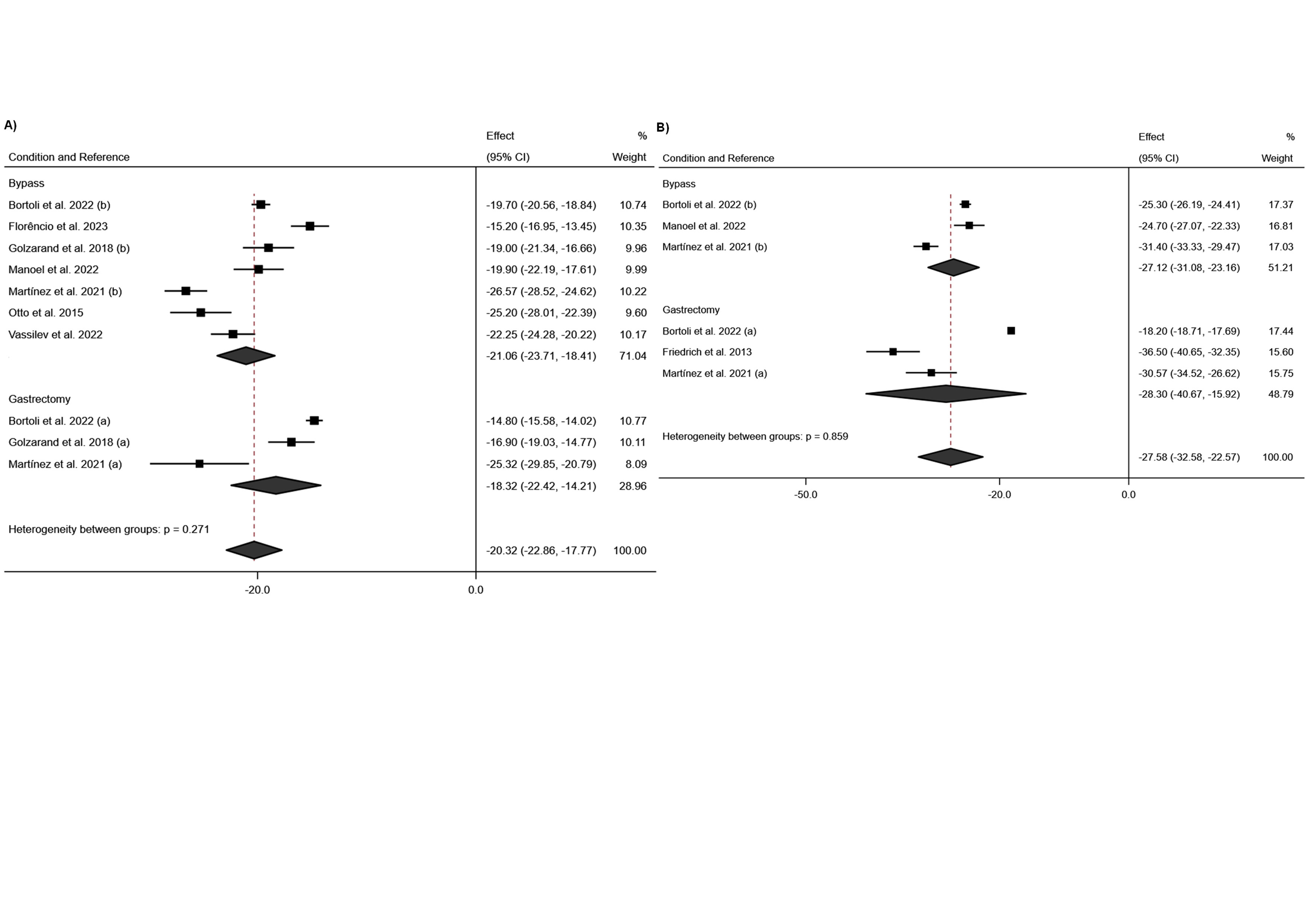

Supplement: Supplementary file 1 [file jcm-13-06784-s001.zip › Figure S4.tif]

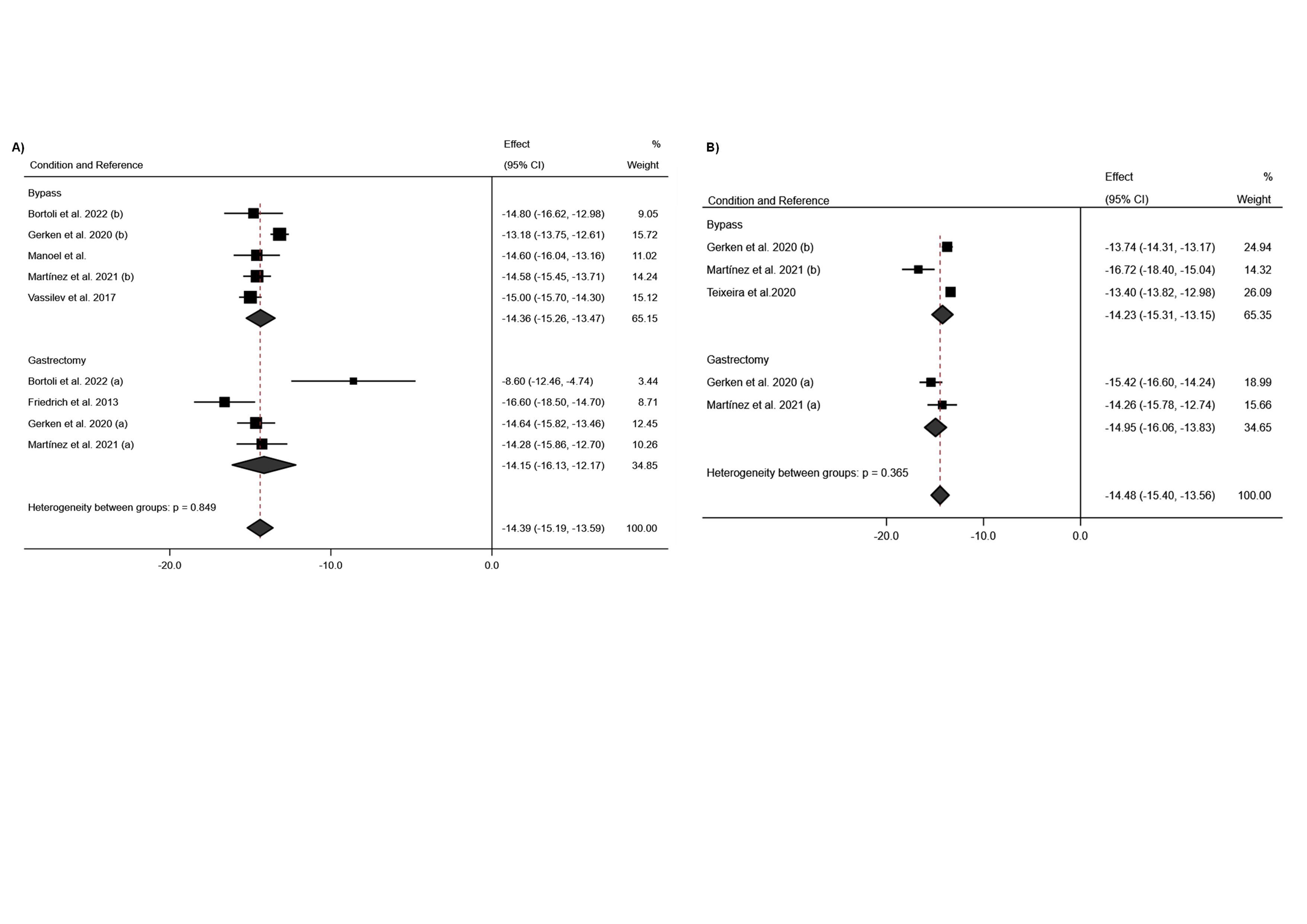

Supplement: Supplementary file 1 [file jcm-13-06784-s001.zip › Figure S5.tif]

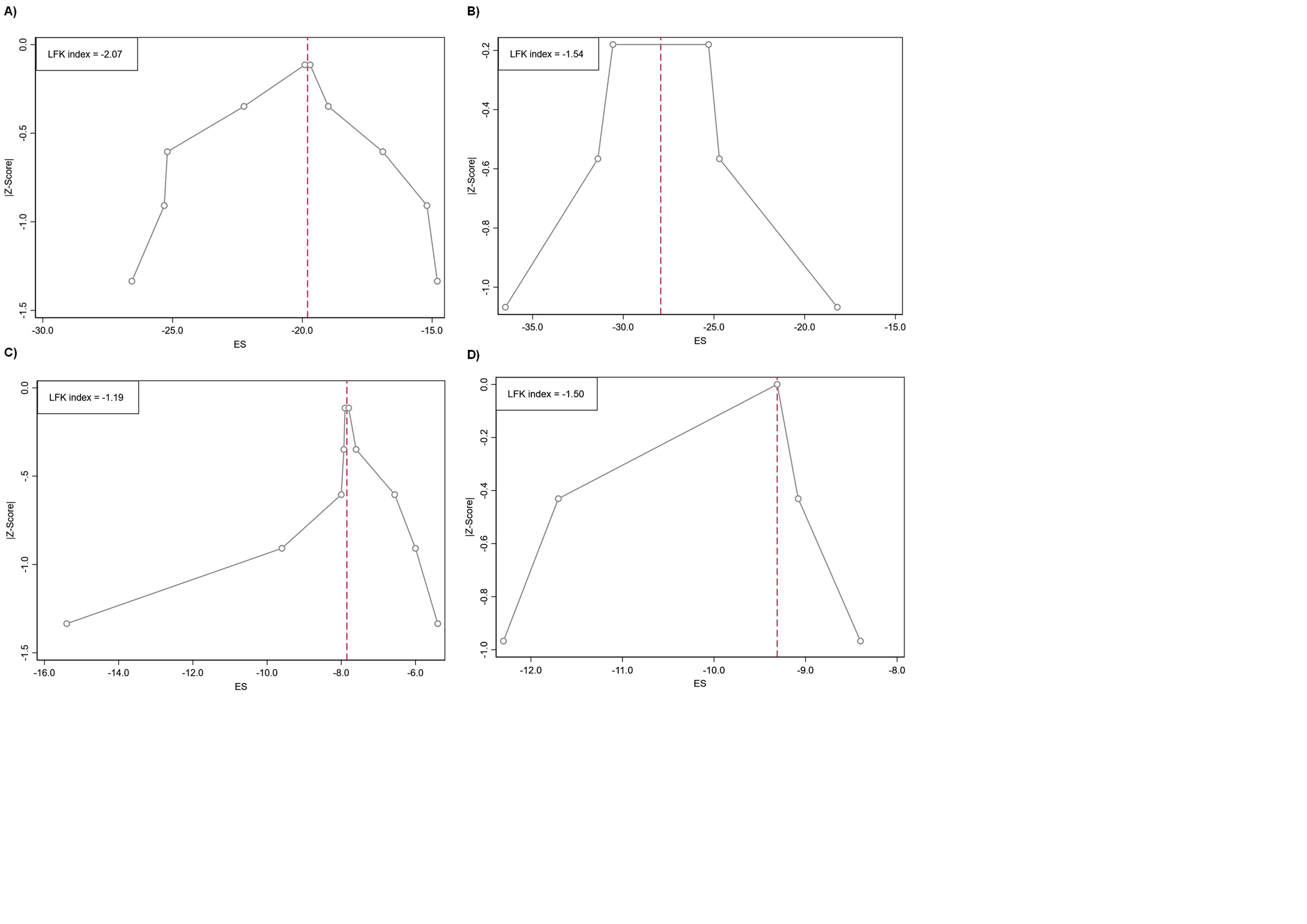

Supplement: Supplementary file 1 [file jcm-13-06784-s001.zip › Figure S6.tif]

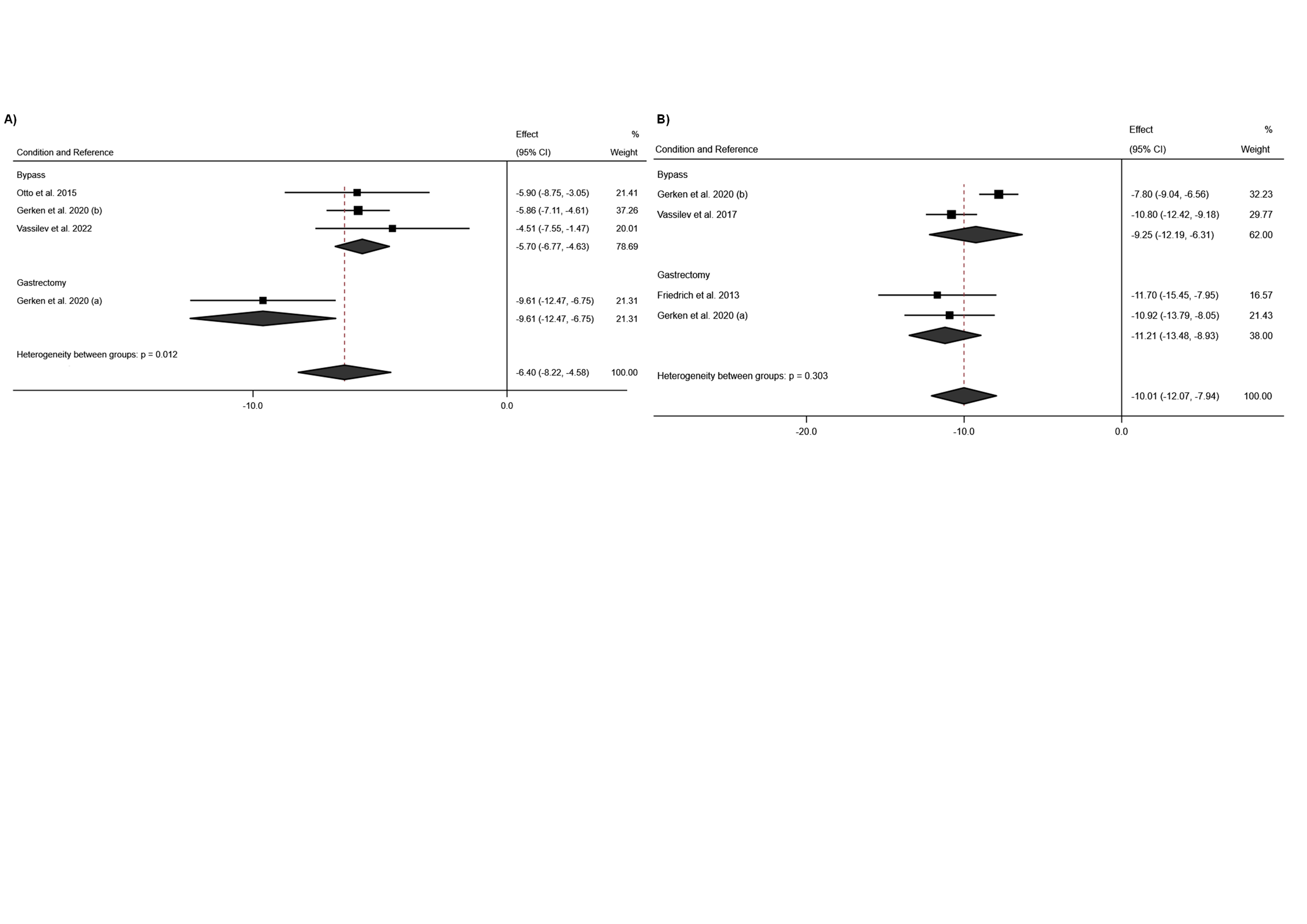

Supplement: Supplementary file 1 [file jcm-13-06784-s001.zip › Figure S7.tif]

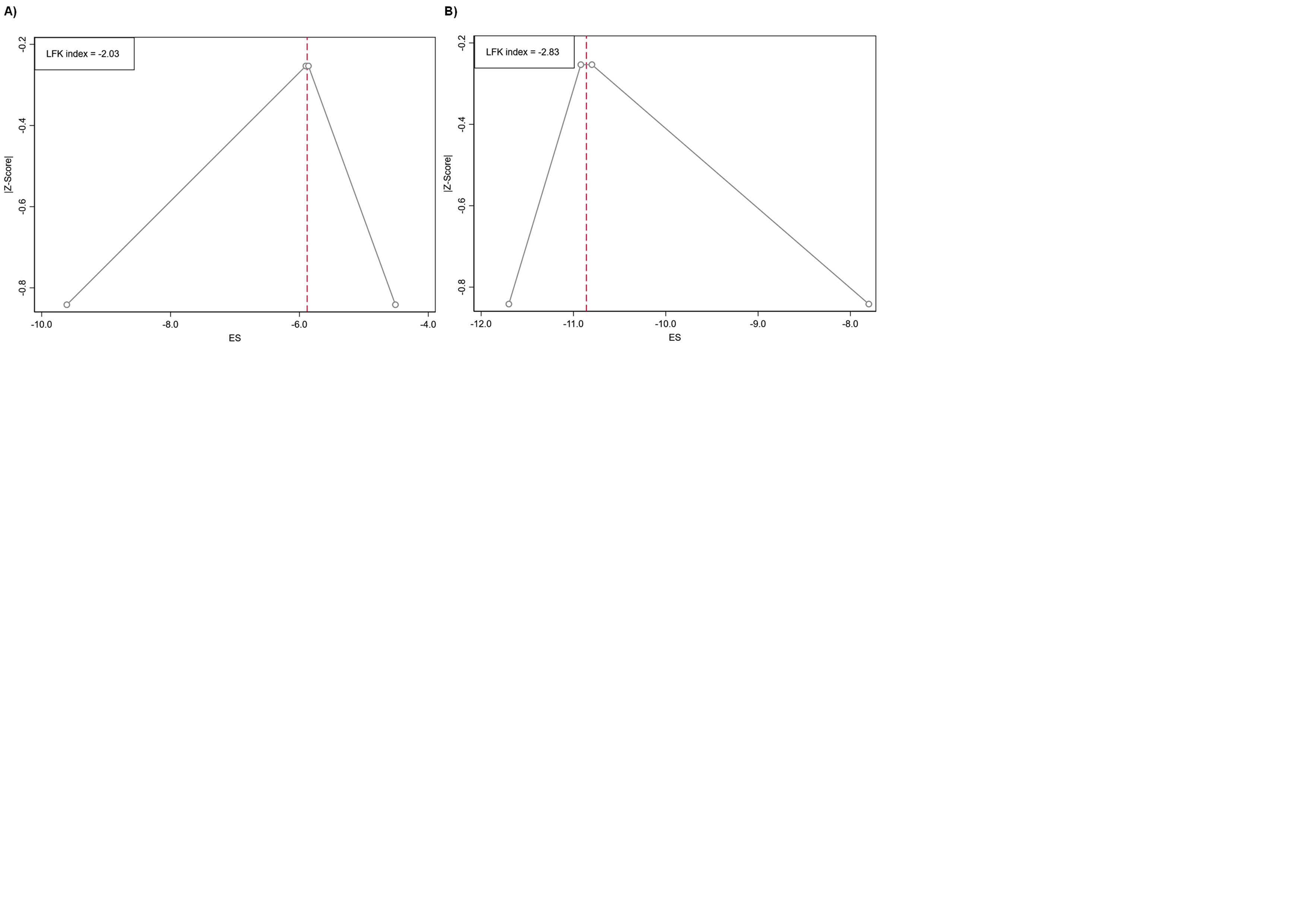

Supplement: Supplementary file 1 [file jcm-13-06784-s001.zip › Figure S8.tif]

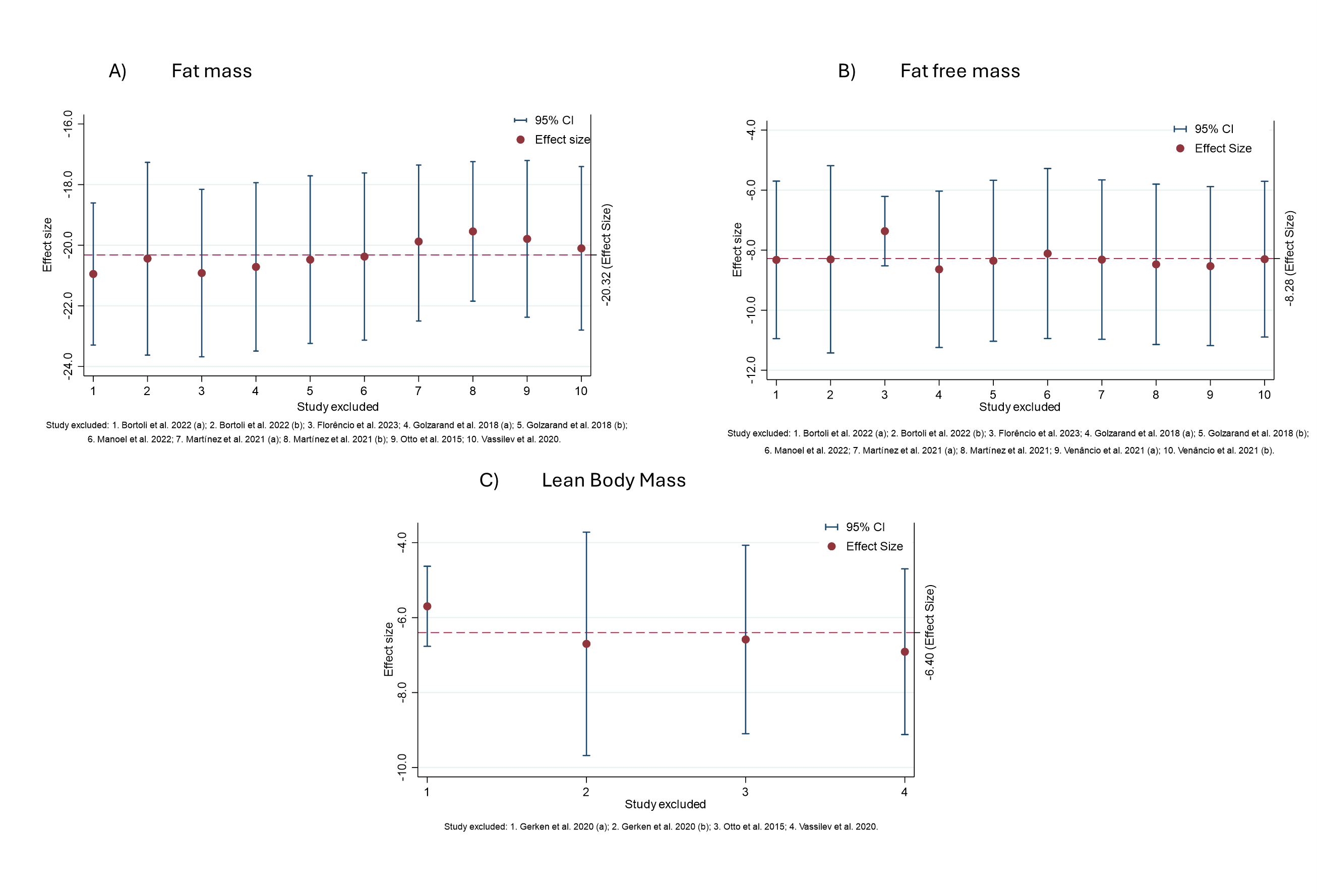

Supplement: Supplementary file 1 [file jcm-13-06784-s001.zip › Figure S9.tif]
